# Defects in tRNA Modification Associated with Neurological and Developmental Dysfunctions in *Caenorhabditis elegans* Elongator Mutants

**DOI:** 10.1371/journal.pgen.1000561

**Published:** 2009-07-10

**Authors:** Changchun Chen, Simon Tuck, Anders S. Byström

**Affiliations:** 1 Department of Molecular Biology, Umeå University, Umeå, Sweden; 2 Umeå Centre of Molecular Medicine, Umeå University, Umeå, Sweden; Huntsman Cancer Institute, United States of America

## Abstract

Elongator is a six subunit protein complex, conserved from yeast to humans. Mutations in the human Elongator homologue, hELP1, are associated with the neurological disease familial dysautonomia. However, how Elongator functions in metazoans, and how the human mutations affect neural functions is incompletely understood. Here we show that in *Caenorhabditis elegans*, ELPC-1 and ELPC-3, components of the Elongator complex, are required for the formation of the 5-carbamoylmethyl and 5-methylcarboxymethyl side chains of wobble uridines in tRNA. The lack of these modifications leads to defects in translation in *C. elegans*. ELPC-1::GFP and ELPC-3::GFP reporters are strongly expressed in a subset of chemosensory neurons required for salt chemotaxis learning. *elpc-1* or *elpc-3* gene inactivation causes a defect in this process, associated with a posttranscriptional reduction of neuropeptide and a decreased accumulation of acetylcholine in the synaptic cleft. *elpc-1* and *elpc-3* mutations are synthetic lethal together with those in *tuc-1*, which is required for thiolation of tRNAs having the 5′methylcarboxymethyl side chain. *elpc-1*; *tuc-1* and *elpc-3*; *tuc-1* double mutants display developmental defects. Our results suggest that, by its effect on tRNA modification, Elongator promotes both neural function and development.

## Author Summary

The efficiency of protein synthesis can be modulated by alterations of various components of the translation machinery. In translation, transfer RNAs act as adapter molecules that decode mRNA into protein and thereby play a central role in gene expression. In the tRNA maturation process, a subset of the normal nucleosides undergoes modifications. Modified nucleosides in the tRNA anticodon region are important for efficient translation. We found that, in the worm *C. elegans*, components of the Elongator complex are required for the formation of a certain set of tRNA modifications in the anticodon region. We observed a reduced efficiency of translation as well as a lower production of neurotransmitters in Elongator mutant worms. Elongator is conserved in eukaryotes, and mutations in a subunit of human Elongator cause a severe neurodegenerative disease, familial dysautonomia (FD). It is unclear in humans whether Elongator acts on the translational level through tRNA modification to regulate neuronal processes. Our observations in *C. elegans*, together with the role of yeast Elongator in translation, show that the function of Elongator in tRNA modification is conserved. Inactivation of Elongator may cause neuronal defects by affecting translation.

## Introduction

Regulation at the level of translation is one important way in which gene activity is controlled in metazoans. Several different mechanisms have previously been identified by which translation can be regulated during development or memory formation [reviewed in 1]. During anterior-posterior patterning of the Drosophila embryo, the translation of *hunchback* mRNA in the posterior region of the embryo is inhibited by binding of a protein complex to the Nanos response element in the hunchback 3′UTR [2]. In *Caenorhabditis elegans*, developmental timing is controlled by the small temporal RNAs, *lin-4* and *let-7*, which act by forming heteroduplexes with their target mRNAs and, at least in some cases, suppressing their translation [3]. Translation efficiency is also regulated by phosphorylation of translational components at the initiation and elongation steps [4],[5]. For example, during memory formation in mice, translation of ATF4 mRNA is regulated by phosphorylation of initiation factor eIF2α [6].

**Figure 1:**
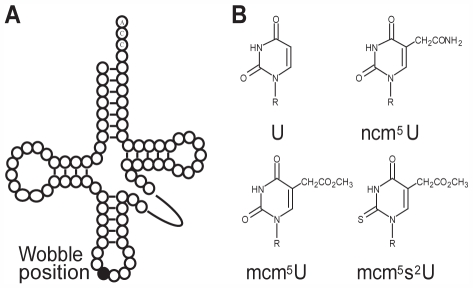
Schematic drawing of the tRNA secondary structure and modified wobble uridines. (A) Secondary structure of tRNA with wobble position shown (•). (B) Wobble uridines can be modified to 5-carbamoylmethyluridine (ncm^5^U), 5-methoxycarbonylmethyl (mcm^5^U) or 5-methoxycarbonylmethyl-2-thiouridine (mcm^5^s^2^U).

Another way in which the efficiency of translation can be modulated is by covalent modification of nucleosides in the anticodons of tRNAs. In the decoding of mRNA, modified nucleosides in the anticodon region, especially position 34 (wobble position) and position 37, have been suggested to be important for restriction or improvement of codon-anticodon interactions [7]–[10]. In *S. cerevisiae*, 25% of the tRNA species are covalently modified by the addition of either carbamoylmethyl (ncm) or methoxycarbonylmethyl (mcm) side chains to the 5′carbon of U_34_ [11]–[14]. A subset of these tRNAs contains a further modification on wobble uridines, addition of a thio group at the 2′position (Figure 1) [11],[13],[14]. *In vivo*, presence of an 5-carbamoylmethyluridine (ncm^5^U), an 5-methoxycarbonylmethyluridine (mcm^5^U) or an 5-methoxycarbonylmethyl-2-thiouridine (mcm^5^s^2^U) improves reading of both A- and G-ending codons [14]–[16].

In *S. cerevisiae*, formation of ncm and mcm side chains present at 5′position of wobble uridines requires the Elongator complex [12], which is composed of six subunits Elp1p – Elp6p [17],[18]. Yeast cells lacking Elongator activity are viable but display multiple defects including those in PolII transcription and exocytosis [16], [18]–[22]. However, these defects all appear to result from a primary defect in tRNA modification [16]. Elongator complex is conserved in eukaryotes and has also been purified from humans [23]. Inactivation of Elongator subunits in multicellular organisms causes multiple defects including those in development, cell proliferation, cell migration and neuron projection [24]–[27]. Recently, Elongator in mice has been reported to acetylate α-tubulin [27]. However, it is presently unclear whether Elongator in higher eukaryotes functions directly in multiple processes or acts on a small number of targets whose absence leads to pleiotropic defects.

Mutations in the human homologue of yeast *ELP1*, *IKBKAP*/*hELP1*, have been shown to cause Familial Dysautonomia (FD), a genetic disorder primarily affecting the sensory and autonomic nerve systems [28]–[30]. Human IKAP/hELP1 protein is part of a complex of six proteins that also contains the human homologues of yeast Elongator proteins [23]. Whether Elongator in humans or other metazoans promotes tRNA modification has not been reported.

The aim of the present study was to investigate the function of the Elongator homologues, ELPC-1 and ELPC-3 in the nematode, *C. elegans*. In particular, we were interested to determine first, whether Elongator in metazoans is required for modification of wobble uridines, and second, whether *C. elegans* could be established as a model to study the role of Elongator in modulating translation within neurons and other tissues. We demonstrate that Elongator is required in *C. elegans* for the formation of modified nucleosides in tRNA, and that Elongator mutants have defects in neurological and developmental processes associated with reduced translation. We believe our results also have important implications for the etiology of FD disease.

## Results

### *C. elegans elpc-1* and *elpc-3* Are Required for Synthesis of mcm^5^ and ncm^5^ Side Chains at Wobble Uridines

**Figure 2:**
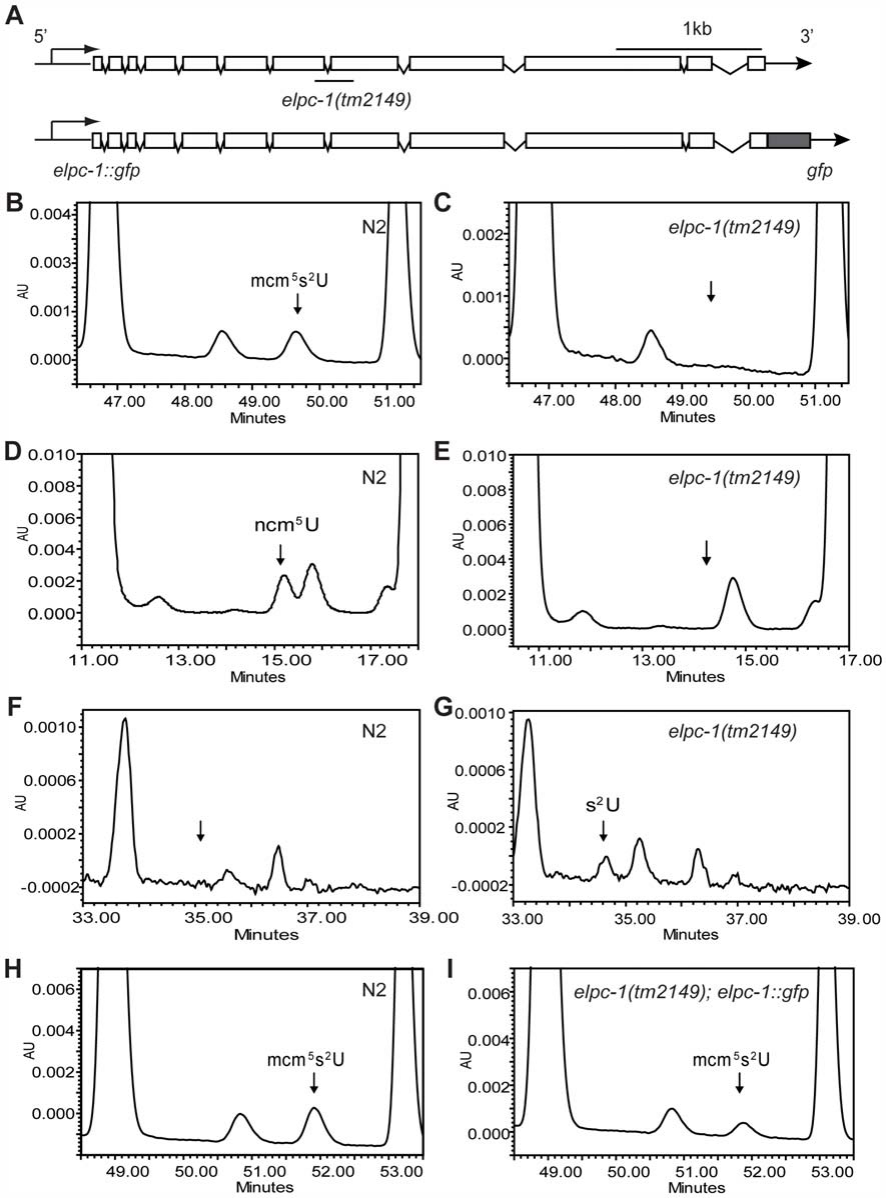
*C. elegans elpc-1* is required for mcm^5^ and ncm^5^ side chain formation at wobble uridines. (A) Schematic drawing of the distribution of exons and introns of *elpc-1*. The exons and introns are depicted as boxes and lines, respectively. At top, the line underneath represents the location of the deletion in *elpc-1(tm2149)*. Below, the structure of the *elpc-1::gfp* transgene. (B–I) Total tRNA isolated from wild-type and *elpc-1(tm2149)* worms was analyzed by HPLC. Wild-type (N2) profiles are shown in the left panels. *elpc-1(tm2149)* and *elpc-1(tm2149)*; *elpc-1::gfp* profiles are in the right panels. Chromatograms were monitored at 254 nm unless otherwise stated. (B,C) The parts of the chromatograms between retention times 46.4 and 51.5 min are displayed. The arrow in C indicates the expected retention time of mcm^5^s^2^U. (D,E) The parts of the chromatograms between retention times 10.5 and 18 min are displayed. The arrow in E indicates the expected retention time of ncm^5^U. (F,G) The parts of the chromatograms between retention times 33 and 39 min are displayed. The arrow in F indicates the expected retention time of s^2^U. These chromatograms were monitored at 314 nm. (H,I) The parts of the chromatograms between retention times 48 and 54 min are displayed.

Searches of the *C. elegans* protein sequence database with the yeast or human Elp1p and Elp3p sequences revealed that *C. elegans* contains single Elp1p and Elp3p homologues, named ELPC-1 and ELPC-3, which are encoded by Y110A7A.16 and ZK863.3 respectively [see Materials and Methods for an explanation of gene nomenclature]. To investigate the function of *elpc-1* and *elpc-3* in *C. elegans*, we used *elpc-1*(*tm2149*) and *elpc-3*(*tm3120*), deletion mutants kindly supplied by S. Mitani of the National Bioresource Project, Japan. The *elpc-1*(*tm2149*) deletion removes 275 bp of sequence spanning parts of exons 7 and 8 (Figure 2A), whereas the *elpc-3*(*tm3120*) removes 356 bp spanning the first half of exon 3 and contains as well an insertion of four nucleotides in the second half of exon 3 (Figure S1A). The *elpc-3*(*tm3120*) deletion removes part of a sequence sharing significant homology to the Radical S-adenosylmethionine (SAM) superfamily [31]. Members of this family of proteins contain an FeS cluster and use S-adenosylmethionine (SAM) to catalyse a variety of radical reactions. The Elp3p Radical SAM domain has been found to be required for iron binding in *Methanocaldococcus jannaschi* [32], and for integrity of the Elongator complex in yeast [33].

In yeast, Elp1p and Elp3p are required for the formation of mcm^5^ and ncm^5^ side chains of modified nucleosides mcm^5^U, ncm^5^U, ncm^5^U_m_ and mcm^5^s^2^U present at the wobble position in tRNA [12]. To determine whether their homologues in *C. elegans*, ELPC-1 and ELPC-3, also function to promote wobble uridine tRNA modification, we examined if the mcm^5^U, ncm^5^U or mcm^5^s^2^U modified nucleosides were present in tRNA isolated from wild-type and *elpc* mutants. Total tRNA isolated from wild-type worms contained ncm^5^U and mcm^5^s^2^U nucleosides (Figure 2B and 2D, Figure S1B, S1D). However, no mcm^5^U was detected (Figure S2D), implying that modification of uridine in *C. elegans* tRNA differs in at least one respect from that in *S. cerevisiae*. In contrast to wild-type worms, no mcm^5^s^2^U or ncm^5^U nucleosides were present in tRNA isolated from *elpc-1*(*tm2149*) mutants (Figure 2C and 2E). Instead, 2-thio uridine (s^2^U) was detected in tRNA isolated from the *elpc-1*(*tm2149*) mutant but not from wild-type worms (Figure 2F and 2G). This nucleoside arose from a failure in the mutant to add the mcm^5^ side chain of the mcm^5^s^2^U nucleoside. The tRNA modification defect in the *elpc-1*(*tm2149*) mutant was rescued by *elpc-1* activity provided by a transgene (Figure 2C and 2I). Thus, like yeast Elp1p, *C. elegans* ELPC-1 is required for the formation of mcm^5^ and ncm^5^ side chains in tRNA. Consistent with the tRNA modification defect in the *elpc-1*(*tm2149*) mutant, tRNA isolated from *elpc-3*(*tm3120*) mutants lacked the mcm^5^s^2^U and ncm^5^U nucleosides and instead contained s^2^U (Figure S1).

Synthesis of the s^2^ group of mcm^5^s^2^U in yeast requires Tuc1p [15], [34]–[38]. The homologue of Tuc1p in *C. elegans* is encoded by open reading frame F29C4.6 [39]. In this paper we will refer the F29C4.6 gene as *tuc-1*. We analyzed tRNA from *tuc-1(tm1297)* mutant worms by HPLC and confirmed that it lacked the mcm^5^s^2^U modification and instead had mcm^5^U, a nucleoside not normally found in *C. elegans* tRNA (Figure S2B, S2C, S2D, S2E). Furthermore, a transgene containing wild-type *tuc-1* DNA restored formation of mcm^5^s^2^U in tRNA (data not shown). Consistently, tRNA isolated from an *elpc-1*(*tm2149*); *tuc-1*(*tm1297*) double mutant lacked both the 5′- and 2′ side-chains of wobble uridines and no ncm^5^U or mcm^5^s^2^U nucleosides were observed (Figure S3).

### An *elpc-1::gfp* Reporter Gene Is Differentially Expressed

**Figure 3:**
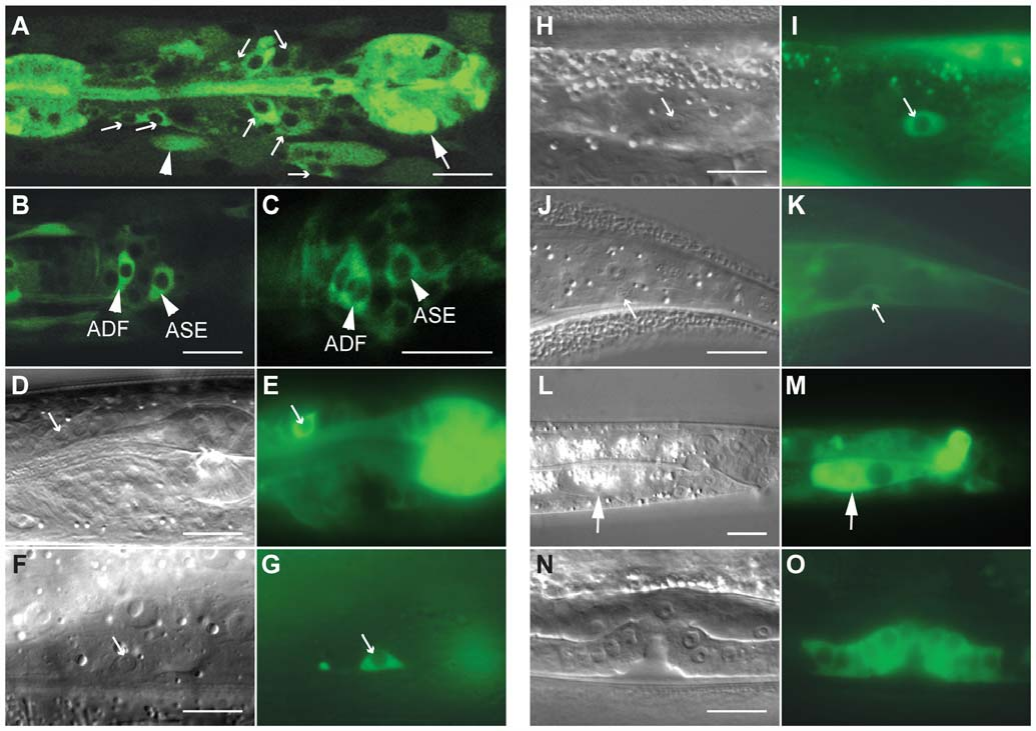
ELPC-1 is differentially expressed in *C. elegans*. (A–C) Confocal fluorescence micrographs of an hermaphrodite of the genotype *elpc-1(tm2149)*; *svEx557*[P*_elpc-1_::elpc-1::gfp*]. The large arrow in A denotes the posterior bulb of the pharynx. The smaller arrows denote sensory neurons in the head. The arrowhead indicates a body muscle. In B and C, specific sensory neurons in the head are indicated. (D–O) Micrographs of *elpc-1(tm2149)*; *svEx557*[P*_elpc-1_::elpc-1::gfp*] worms viewed with either Nomarski differential contrast (DIC) (D,F,H,J,L,N) or fluorescence (E,G,I,K,M,O) optics. The arrows in D and E indicate an ASK sensory neuron; in F and G, an HSN; in H and I, a CAN cell; in J and K, a PLM neuron; in L and M, a cell in the intestine. The green fluorescence in O is from cells in the developing vulva. Scale bars denote 10 microns.

To investigate the expression pattern of *C. elegans* ELPC-1 in various tissues, we examined worm strains harboring a transgene encoding functional, full length ELPC-1 protein fused to GFP. The transgene contained 435 bp of the promoter region and all 11 introns (Figure 2A). The transgene rescued the tRNA modification defect in the *elpc-1* mutants (Figure 2C and 2I). The fusion protein encoded by the transgene was preferentially detected in several tissues including the nervous system (Figure 3). However, its presence was not uniform. Within the nervous system, ELPC-1::GFP was seen predominantly in a pair of neurons that control egg-laying, the HSNs (Figure 3F and 3G), and in chemosensory neurons in the head (Figure 3A–3E). Within the latter class of neurons, the ELPC-1::GFP level was particularly high in the ASE, ADF and ASK pairs of neurons (Figure 3B–3E. For nomenclature of neurons, see Materials and Methods). Expression was seen both within the cell bodies (Figure 3B) and along the entire lengths of the neuronal processes (data not shown). Outside of the nervous system, a strong ELPC-1::GFP signal was seen in the pharynx (the feeding organ) (Figure 3A) and the vulva (Figure 3N and 3O), part of the egg-laying apparatus in the hermaphrodite. In all animals examined, ELPC-1::GFP expression was also seen in the two CAN cells (Figure 3H and 3I), which are associated with the excretory canals and are required for proper function of the excretory system. In all cells in which ELPC-1::GFP was seen, fluorescence was restricted to the cytoplasm (Figure 3A). The ELPC-3::GFP fusion was expressed in the same set of cells (data not shown).

### Wobble Uridine tRNA Modifications Promote Efficient Translation

In *S. cerevisiae*, defects in wobble uridine tRNA modification are associated with reduced translation efficiency [14]–[16],[40]. The yeast *elp3 tuc1* double mutant, in which modifications at both the 5′and 2′positions of the uridine moiety are absent, is lethal [15]. To investigate the influence of wobble uridine modifications on the efficiency of translation in *C. elegans*, we examined the effect of *elpc-1*, *elpc-3* and *tuc-1* mutations on β-galactosidase expression in worms harboring a *lacZ* transgene driven by heat shock-responsive elements from the *hsp16-1* gene. The induction of *lacZ* mRNA upon heat shock was not reduced in strains lacking wild-type *elpc-1*, *elpc-3* or *tuc-1* gene activity, or in *elpc-1*; *tuc-1* double mutant worms (Table 1). However, β-galactosidase activity was 28% lower in protein extracts from heat shocked *elpc-1*; *tuc-1* double mutants than in those from wild-type worms subjected to the same heat shock regime (Table 1). A modest (∼14–18%) but significant reduction in β-galactosidase activity was also seen *elpc-1*(*tm2149*), *elpc-3*(*tm3120*) or *tuc-1*(*tm1297*) single mutant worms (Table 1).

**Table 1:** Lack of Wobble Uridine tRNA Modification Affects Translation Efficiency.

Strains	β-gal activity	*lacZ* mRNA/*tbb-2* mRNA
N2^a^	312.57±17.15 (100.0%)	1.00±0.09
*elpc-1(tm2149)* a	267.84±19.84* (85.7%)	0.90±0.05
*elpc-3(tm3120)* a	257.39±9.37* (82.3%)	0.92±0.07
*tuc-1(tm1297)* a	264.54±14.66* (84.6%)	0.89±0.12
N2^b^	322.43±4.57 (100.0%)	1.00±0.08
*elpc-1(tm2149);tuc-1(tm1297)* b	233.40±6.34** (72.4%)	1.28±0.22

a The strains were grown at 20°C before heat-shock at 33°C. The difference of β-gal activity (mean±SD) between N2 and *elp-1(tm2149)*, *elp-3(tm3120)* or *tuc-1(tm1297)* was analyzed by student's t test (*p<0.01). For the QRT-PCR data (mean±SD), the *lacZ* mRNA was normalized against *tbb-2* and *ubc-2* mRNA (data not shown). In the table are the ratios between *lacZ* and *tbb-2* mRNA. There is no reduction on mRNA level in the mutant strains.

b The strains were grown at 15°C before heat-shock at 33°C. The reduction of β-gal activity (mean±SD) in *elp-1(tm2149)*; *tuc-1(tm1297)* is significant compared to the wild type (**p<0.001). The *lacZ* mRNA level (mean±SD) was also normalized against *tbb-2* and *ubc-2* mRNA (data not shown).

**Figure 4:**
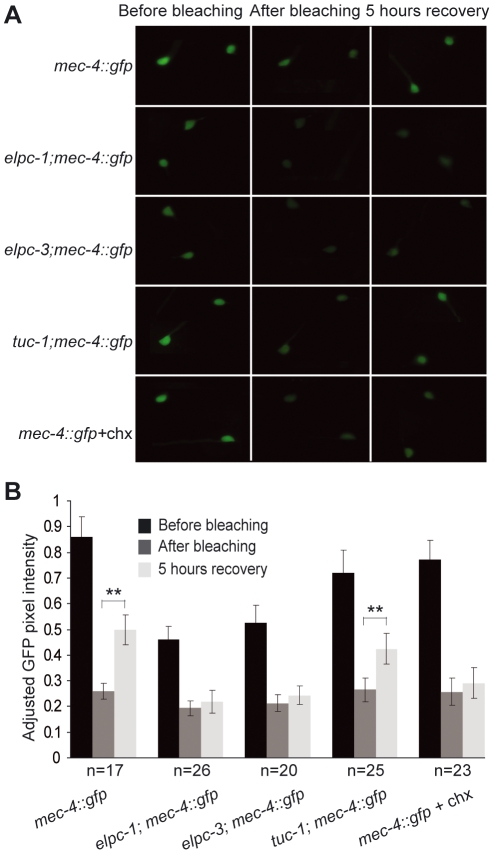
The *elp-1(tm2149)* and *elpc-3(tm3120)* mutants are defective in fluorescence recovery after photobleaching. (A) Images of *mec-4::gfp* reporter in wild type, *elpc-1*, *elpc-3* and *tuc-1* backgrounds before photobleaching (left), after photobleaching (middle) and 5 hours recovery (right). The images at the bottom are of the *mec-4::gfp* reporter strain treated with the cycloheximide. (B) Quantification of GFP pixel intensities before photobleaching, after photobleaching and 5 hours recovery. The number of worms examined of each strain is given under the graph. Error bars represent standard deviations. Two asterisks indicate a significant fluorescence recovery after 5 hours incubation by student's t test (**p<0.001).

To monitor cell and tissue specific protein synthesis, we used an established technique, fluorescence recovery after photobleaching (FRAP) [41]. The rate of protein synthesis in different cells and tissues was measured using GFP reporters. We used *gcy-5::gfp* and *mec-4::gfp* which are expressed in ASER and 6 touch cell neurons respectively, and *myo-3::gfp* which is expressed in the body wall muscle. In all reporter fusions examined, photobleached wild type animals showed a significant recovery of GFP signal within 5 hours (Figure 4, Figure S4). However, animals with the *elpc-1*(*tm2149*) or *elpc-3*(*tm3120*) mutations had a slower GFP signal recovery, indicating a reduced rate of protein synthesis (Figure 4, Figure S4). Cycloheximide, an inhibitor of translation, was used to confirm that the recovered GFP signal was due to newly synthesized protein. In animals treated with cycloheximide, no significant recovery of GFP signal was observed (Figure 4, Figure S4). Together, these experiments demonstrate that an absence of uridine modification in tRNA is associated with a reduction in translation efficiency in *C. elegans*.

### *elpc-1* and *elpc-3* Mutants Show Defects in Neuronal Function

**Figure 5:**
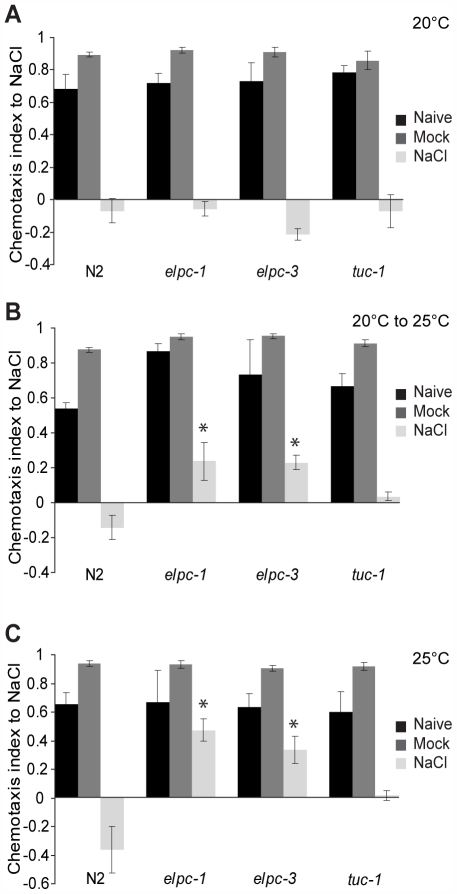
The *elp-1(tm2149)* and *elpc-3(tm3120)* mutants show a salt chemotaxis learning defect. For each strain there are three conditions. ‘NaCl’ indicates worms that were preconditioned on a plate containing 100 mM NaCl for 4 hours prior to the chemotaxis assay. ‘Mock’ indicates that worms were pretreated on NaCl-free plate for 4 hours before assay. ‘Naive’ indicates worms that were assayed without any preconditioning. The chemotaxis index after 30 min of assay is displayed. The assay was repeated four times. Error bars denote standard deviations. Asterisk indicates a significant difference from wild type N2 (*p<0.01 by student's t test). (A) Young adult worms that had been raised at 20°C. (B) Temperature shifted animals. Synchronized eggs were grown at 20°C to the 2^nd^ larval stage (L2). L2 larvae were then shifted to 25°C and cultured until they had become young adults. (C) Young adult worms from a strain that had been maintained at 25°C for several generations.

*elpc-1* and *elpc-3* single mutants were viable and fertile and they were able to move normally on the bacterial lawn. Furthermore, the chemosensory neurons in which ELPC-1::GFP and ELPC-3::GFP are strongly expressed are present at their normal positions and have normal morphology (Figure S5). Among these neurons (Figure 3A–3E), the ASE pair of sensory neurons is required for experience-dependent behaviors elicited by different salt concentrations [42]. Wild-type worms normally chemotax towards NaCl. However, pre-incubation in normal salt concentrations in the absence of nutrients elicits an aversion response to NaCl when worms are subsequently tested in chemotaxis assays [43]. In this salt learning assay, worms that have grown at normal salt concentrations and in the presence of abundant nutrients are first starved for four hours in the presence or absence of salt and then assayed for their chemotactic response to NaCl. Since we observed strong expression of ELPC-1 and ELPC-3 in ASE neurons (Figure 3B and 3C, data not shown), we tested *elpc-1* and *elpc-3* mutants in a salt learning assay. At 20°C, the mutants behaved as wild type (Figure 5A). At 25°C, wild-type worms exposed to 100 mM NaCl in the absence of nutrients moved away from NaCl, whereas *elpc-1* or *elpc-3* mutants treated in the same way continued to chemotax towards the NaCl (Figure 5C). In the *elpc-1* mutant, this defect was partially rescued by the *elpc-1::gfp* construct (Figure S6). Thus, *C. elegans elpc-1* and *elpc-3* are required for an experience-dependent change in behavior. In contrast, in *tuc-1* mutant worms no statistically significant changes were observed (Figure 5).

Ablation of the ASE neurons leads to an inability to chemotax towards certain water-soluble compounds including Na^+^, Cl^−^, lysine and biotin [44]. *elpc-1* and *elpc-3* mutants were able to chemotax both to water soluble and volatile compounds at all temperatures tested (Figure S7). When *elpc-1* or *elpc-3* mutants were grown at 20°C to the time at which the chemosensory neurons have developed and then shifted to 25°C, salt learning was abnormal (Figure 5B). Together, these observations suggest that the salt learning defect seen in *elpc-1* and *elpc-3* mutants is not caused by a defect in the development of the ASE chemosensory neurons or in their ability to detect salt.

### The Level of a Neuropeptide::GFP Reporter in Neurons Is Reduced in *elpc* Mutants

**Figure 6:**
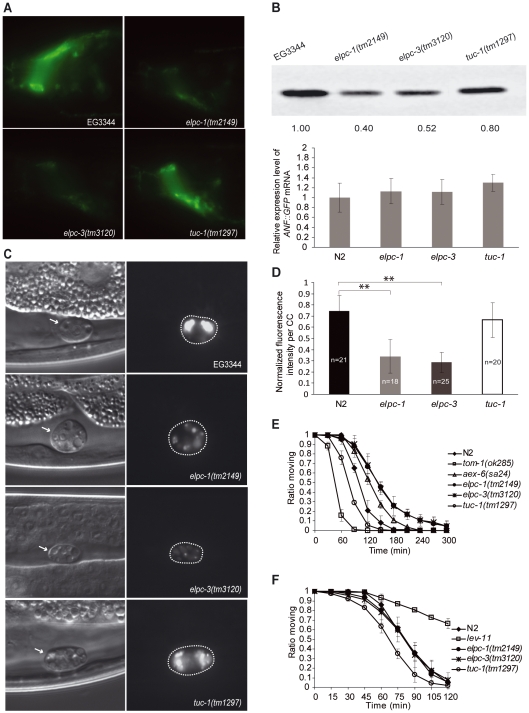
Neurons in the *elpc-1*(*tm2149*) and *elpc-3*(*tm3120*) mutants show reduced production of neuropeptide. (A) Fluorescence micrographs showing the nerve ring in worms harboring a transgene encoding ANF::GFP. (B) At top, western blot of protein extracts from worms of the indicated genotypes that contained the ANF::GFP transgene. The same amount of protein was loaded in each lane. The blot was probed with an antibody against GFP. Below, *ANF::GFP* transcripts were quantified by Real-time PCR. No significant difference was observed in the levels of *ANF::GFP* mRNA (Student's t test, p>0.05), which were normalized to *tbb-2* mRNA. (C) Micrographs showing coelomocytes in worms carrying the *ANF::GFP* transgene. Those on the left were viewed with DIC optics. Those on the right are of the same worms viewed with fluorescence optics. Dashed lines indicate the locations of the coelomocytes. Note that the intensity of GFP fluorescence in EG3344 and *tuc-1(tm1297)* coelomocytes is higher than that in *elpc-1*(*tm2149*) and *elpc-3*(*tm3120*) mutant worms. (D) Graph showing the normalized pixel intensities of confocal images of coelomocytes (CC). The number of coelomocytes measured for each strain is shown on the bar. The strongest pixel intensity per coelomocyte of ANF::GFP in any worm tested was arbitrarily set to 1. Error bars represent standard deviations. Two asterisks indicate the significant difference from control worms by student's t test (**p<0.001). (E) Aldicarb sensitivity assays. The proportion of worms still able to move is plotted against time for the six different genotypes. N2 is the wild-type control; *aex-6*(*sa24*) is a strain previously shown to display increased resistance to aldicarb, and *tom-1*(*ok285*) is hypersensitive to aldicarb. (F) Levamisol sensitivity assays were performed in the same way as aldicarb assays. N2 is the wild-type control. *lev-11* is a strain previously shown to be resistance to levamisol.

Since neuronal function in metazoans is known to be dependent upon the ability to synthesize and secrete neurotransmitters and neuropeptides, we tested whether these processes were abnormal in *C. elegans elpc-1* and *elpc-3* mutants. One established assay for examining the synthesis and secretion of neuropeptides involves a heterologous fusion protein, ANF::GFP. The prodomain of a preproANF–GFP fusion protein can be used as a reliable fluorescent reporter of dense-core vesicle transport and exocytosis in rat PC12 cells, as well as in *D. melanogaster* and *C. elegans* neurons [45]–[47]. In *C. elegans*, ANF::GFP is secreted by neurons into the pseudocoelomic space from where it is rapidly cleared by three pairs of coelomocytes [47]. In *elpc-1* and *elpc-3* mutants, we observed a reduced accumulation of ANF::GFP in coelomocytes (Figure 6C and 6D), which could be caused by either less synthesis or reduced secretion of ANF::GFP from neurons. In both wild-type and *elpc* mutant worms carrying the ANF::GFP transgene, the fusion protein was visible in neurons, but the GFP signal was weaker in *elpc* mutants that was also reflected by western blot (Figure 6A and 6B). As there was no significant reduction of ANF::GFP mRNA in *elpc* mutants, the lower production of ANF::GFP was at the posttranscriptional level (Figure 6A and 6B).

To investigate whether *elpc-1* and *elpc-3* also affected extracellular levels of a neurotransmitter, we examined whether the mutants showed increased resistance to aldicarb, an inhibitor of acetylcholinesterase present in the synaptic cleft. Wild-type worms exposed to aldicarb immediately hypercontract and then die after a few hours because they are unable to reduce synaptic levels of acetylcholine secreted by neurons [48]. Mutants with reduced acetylcholine-mediated signaling are partially or completely resistant to the drug. Aldicarb-resistant mutants fall into two classes, those that have pre-synaptic defects resulting in reduced synthesis or secretion of acetylcholine and those in which the fault lies in the post-synaptic neurons [49]. *elpc-1* and *elpc-3* mutants showed greater resistance to aldicarb than that displayed by *aex-6*(*sa24*) (Figure 6E), which has been described previously as being partially resistant to the drug [50]. *elpc-1(tm2149)* mutant harboring the *elpc-1::gfp* transgene on an array behaved as wild type in the aldicarb assay (Figure S8). *elpc-1* and *elpc-3* mutant worms showed normal response to levamisole (Figure 6F), which activates the post-synaptic acetylcholine receptor [49], suggesting a defect in the pre-synaptic compartment. These results suggest that either less acetylcholine is produced in the neurons or less acetylcholine is released from the neurons in the *elpc-1* and *elpc-3* mutants.

### *C. elegans elpc-1* and *elpc-3* Mutants Have Normal Levels of Acetylated α-tubulin

Recently it was shown that mouse ELP3 protein can acetylate α-tubulin *in vitro* [27]. Thus one possibility is that the neural defects seen in mice with reduced Elongator activity is caused by aberrant α-tubulin function. Acetylation of α-tubulins in a wide variety of species occurs on a conserved lysine residue at position 40. In *C. elegans*, there is a single α-tubulin with a lysine at this position, MEC-12 [51]. To investigate whether Elongator in *C. elegans* promotes acetylation of α-tubulin, we examined acetylation in *elpc-1* or *elpc-3* mutants. As previously reported, an antibody that recognizes lysine 40-acetylated α-tubulin in various species, 6-11B-1, could detect the residue in extracts from wild-type worms but not those from the *mec-12*(*e1607*) mutant. However, we observed no reduction in the levels of acetylated MEC-12 in *elpc-1* or *elpc-3* mutants (Figure S9A). Furthermore, unlike *elpc-1* or *elpc-3* mutants, *mec-12*(*e1607*) is not aldicarb resistant (Figure S9B).

### Synthetic Effects in *elpc-1*; *tuc-1* Double Mutants Indicate a Role for ELPC-1, ELPC-3, and TUC-1 in Development

**Figure 7:**
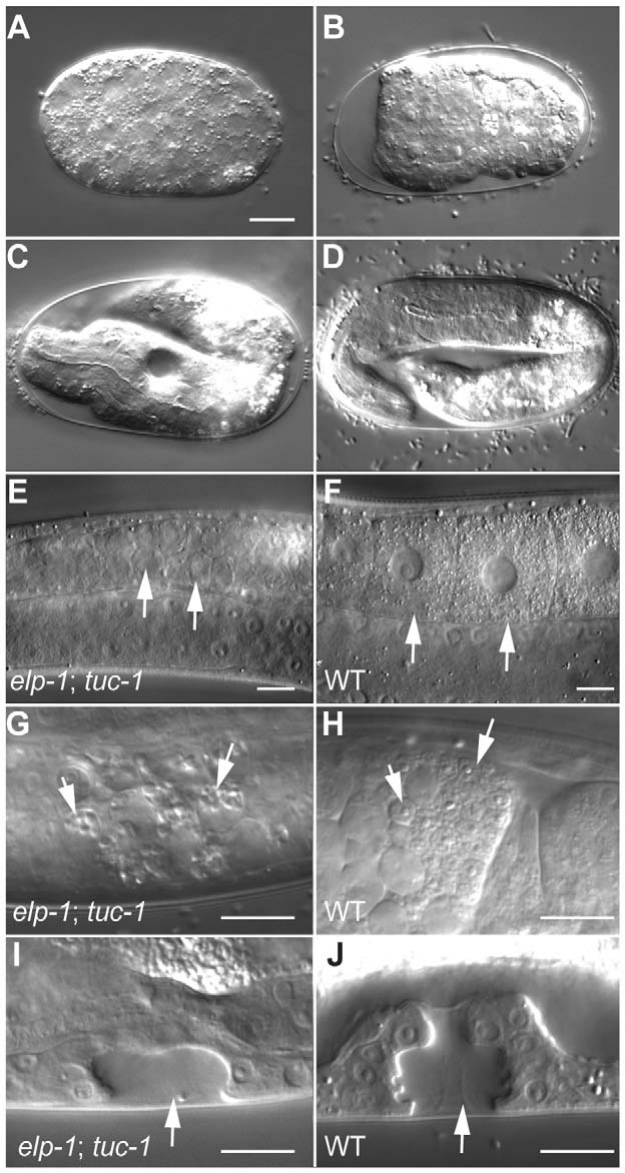
Defects seen in temperature-shifted *elpc-1*; *tuc-1* double mutants. Micrographs of eggs and larvae viewed with Nomarski DIC optics. (A–D) Embryos arrested prior to (A), during (B,C) or after (D) morphogenesis. (E,F,G,H) Parts of the germline in young adult hermaphrodites. The arrows in E and F indicate oocytes. Note that those in the *elpc-1*; *tuc-1* worm have not matured. The arrows in G and H denote sperm. Those in the *elpc-1*; *tuc-1* worm have grossly abnormal morphology. (I,J) The vulva during the L4 stage. In the *elpc-1*; *tuc-1* double mutant, fewer cells are present and morphogenesis of the vulva to form the tube through which the eggs are laid is abnormal. Scale bars denote 10 microns.

In humans, *IKBKAP*/*hELP1* expression is not confined to the nervous system but is also seen in many other cell types [29],[52],[53]. In *C. elegans*, we also observed ELPC-1::GFP expression in several non-neuronal tissues (Figure 3). However, in an otherwise wild-type genetic background, although they grow slower than wild-type and had reduced fertility at 25°C (Table 2), the development of *elpc-1* or *elpc-3* mutants is not grossly disturbed. In yeast, *elp1* and *elp3* deletion strains are also viable. However, yeast cells lacking both *ELP3* and *TUC1*, which therefore lack both mcm^5^ and s^2^ groups of tRNAs having the nucleoside mcm^5^s^2^U_34_, are not viable [15]. In the course of analyzing *elpc-1*; *tuc-1* double mutant worms, we observed that the strain could be propagated at 15°C but not at 25°C. The *elpc-1*(*tm2149*); *tuc-1*(*tm1297*) double mutant hermaphrodites raised at 15°C for different periods of time were shifted to 25°C and then examined both for their own development and also for their ability to give rise to viable progeny. When 4^th^ larval stage (L4) hermaphrodites were shifted to 25°C, they continued to develop and became fertile adults. However, the eggs they laid arrested development during embryogenesis (Figure 7A–7D). The arrest did not occur at one specific embryonic stage but rather at different stages in different embryos. Some embryos were arrested either prior to enclosure with relatively few cells (<100 cells) (Figure 7A); or at the 3-fold stage (Figure 7D). However, the majority were arrested during or immediately after morphogenesis (Figure 7B and 7C). Similar defects were seen in *elpc-3*; *tuc-1* double mutants (Figure S10). Thus ELPC-1, ELPC-3 and TUC-1 likely function at multiple times during embryogenesis. No synthetic defects were seen in *elpc-1*; *elpc-3* double mutants, suggesting that Elongator function is abolished in both *elpc-1* and *elpc-*3 single mutants.

**Table 2:** *elpc-1(tm2149)*, *elpc-3(tm3120)* and *tuc-1(tm1297)* Worms Display Temperature Sensitive Fecundity and Life Cycle Defects.

	Number of eggs produced^a^		Length of reproductive cycle (hours)^b^	
	20°C	25°C	20°C	25°C
N2	255±36 (100.0%)	172±28 (100.0%)	68.9±1.2 (100.0%)	54.6±1.1 (100.0%)
*elpc-1(tm2149)*	303±77 (118.8%)	42±13** (24.4%)	80.3±0.9** (116.5%)	67.8±1.1** (124.2%)
*elpc-3(tm3120)*	292±48 (114.5%)	56±16** (32.6%)	81.2±1.1** (117.9%)	65.5±2.3** (120.0%)
*tuc-1(tm1297)*	274±62 (107.5%)	44±33** (25.6%)	78.5±1.7** (113.9%)	64.7±4.7** (118.5%)

a The total numbers of progeny of 20 worms of each genotype were determined. Asterisks represent significant difference from wild type N2 (***p<0.0001 by student's t test).

b The time between an egg being laid and the worm it gave rise to producing progeny was measured for 10 worms of each genotype (mean±SD, **p<0.001 by student's t test).

Temperature shift experiments with 1^st^ or 2^nd^ stage larvae (L1 or L2) also indicated a role for ELPC-1, ELPC-3 and TUC-1 in development of the vulva and for generation of germ cells. When L1 or L2 larval hermaphrodites containing both the *elpc-1*(*tm2149*) and *tuc-1*(*tm1297*) mutations were raised at 15°C and shifted to 25°C, they developed to become small sterile adults. Inspection of the shifted animals at high magnification indicated that vulval development was invariably abnormal (Figure 7I and 7J, Figure S10I, S10J). In wild-type worms, the three progenitors of the vulva, P5.p, P6.p and P7.p are induced to adopt vulval fates: they divide to give rise to 22 cells that together form a tube through which the eggs are laid in adult hermaphrodites. In the temperature-shifted *elpc-1*; *tuc-1* and *elpc-3*; *tuc-1* double mutants, the divisions of P5.p, P6.p and P7.p failed to occur properly and significantly fewer vulval cells were formed (Figure 7I and 7J, Figure S10I, S10J). At the L3 stage, when the vulval developmental fates are induced, expression of the *elpc-1::gfp* reporter was upregulated in P5.p, P6.p and P7.p as well as in their immediate descendants (Figure S11), suggesting that one or more of the signaling pathways mediating vulval cell fate specification controls *elpc-1* expression. Inspection of the gonads of the temperature shifted double mutants revealed that the overall organization of the germline was relatively normal (data not shown). However, the oocytes completely failed to mature (Figure 7E and 7F, Figure S10E, S10F); the sperm were highly vacuolated and grossly abnormal (Figure 7G and 7H, Figure S10G, S10H). These observations imply that *elpc-1* and *elpc-3* also function in development of non-neuronal tissues.

The developmental defects in the *elpc-1*; *tuc-1* double mutant were rescued by extrachromsomal arrays harboring the *elpc-1::gfp* transgene. When *elpc-1*(*tm2149*); *tuc-1*(*tm1297*) double mutant hermaphrodites raised at 15° were allowed to lay eggs at this temperature for two hours and the eggs subsequently shifted to 25°, the progeny invariably arrested either during embryogenesis or during early larval stages (n = 65). However, 60% (n = 40) of *elpc-1(tm2149)*; *tuc-1(tm1297)*; *svEx808*[*elpc-1::gfp* P*unc-122::gfp*] embryos raised grew to become adults with normal vulval development. 15% of these adults gave rise to some live larval progeny indicating partial rescue of both the germline defect and the requirement during early embryogenesis. A second array, *svEx806*[*elpc-1::gfp* P*unc-122::gfp*] also rescued although not quite as efficiently: 40% of embryos grew to become adults.

## Discussion

Here we show that the *elpc-1* and *elpc-3* genes, homologues to yeast *ELP1* and *ELP3*, are required for formation of the ncm^5^ and mcm^5^ side chains present in the wobble nucleosides, ncm^5^U and mcm^5^s^2^U in *C. elegans* tRNA. Worms with mutations in *elpc-1* or *elpc-3* show a defect in a salt learning assay, associated with reduced expression of neuropeptide and slow accumulation of acetylcholine in the synaptic cleft. *elpc-1::gfp* and *elpc-3::gfp* reporters are strongly expressed in certain sensory neurons including ASE, required for salt learning. *elpc-1* and *elpc-3* mutant phenotypes are strongly exacerbated by mutations in *tuc-1*, which is required for the formation of 2-thio group in the mcm^5^s^2^U modified wobble nucleosides.

### The Role of ELPC-1 and ELPC-3 in tRNA Modification

Although a requirement for the Elongator complex for the modification of wobble uridines in yeast tRNA is well documented [12], studies on the role of Elongator in this process in metazoans have not been previously reported. Our results demonstrating that ELPC-1 and ELPC-3 are required for the addition of mcm^5^ and ncm^5^ side chains to uridine residues in *C. elegans* tRNA imply that Elp1p and Elp3p function has been conserved in evolution. Our results also confirm, however, that differences exist in tRNA modification in eukaryotes. In yeast there are 13 tRNA species with a uridine at the wobble position. Of these, eleven contain the nucleoside ncm^5^U, ncm^5^U_m_, mcm^5^U or mcm^5^s^2^U [11]–[14]. In our analysis of *C. elegans* wild-type tRNAs, we found ncm^5^U and mcm^5^s^2^U but not mcm^5^U. This observation is consistent with an earlier investigation showing that mcm^5^U is not present in tRNAs isolated from calf liver [54]. For example, nucleoside 34 in 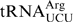 from yeast has mcm^5^U [55], whereas that from calf liver has mcm^5^s^2^U [56]. These findings suggest that mcm^5^U might be absent from tRNAs in metazoans.

In yeast, Elongator was suggested to participate in three distinct cellular processes: transcriptional elongation, polarized exocytosis and formation of modified wobble uridines in tRNA [12],[21],[22]. Strong genetic evidence was provided that the pleiotropic phenotypes seen in yeast, including those in transcription and exocytosis, were caused by a translational dysfunction due to lack of mcm^5^ and ncm^5^ side chains at wobble uridines [16]. This suggests that the physiological relevant role of Elongator complex in this organism is in the formation of modified nucleosides in tRNA, *i.e.* wobble uridine tRNA modification is crucial for the translation of mRNAs that encode proteins important for transcriptional elongation and polarized exocytosis. Cellular localization studies primarily placed Elongator subunits in the cytosol in yeast, mouse and human cells [22], [23], [27], [57]–[60]. As modifications in the anticodon region normally take place in the cytosol [61], such a localization is consistant with a role in wobble uridine modification. In *C. elegans*, we did not observe any fluorescence of ELPC-1::GFP in the nucleus suggesting that Elongator in this organism functions in the cytosol.

### Translation Is Less Efficient in *elpc-1* and *elpc-3* Mutants

In *elpc-1* and *elpc-3* mutants, we observe reduced expression of an ANF::GFP neuropeptide reporter. Given that ANF::GFP mRNA levels are normal in the mutants, the reduction in ANF::GFP accumulation could in principle be explained either by increased degradation of the protein or by decreased translation. Since tRNAs are intimately involved in protein synthesis, we believe it more likely that ELPC-1 and ELPC-3 affect ANF::GFP levels by promoting translation. Further evidence indicating a role for Elongator in translation is that the recovery of GFP signals after photobleaching in strains with *gcy-5::gfp*, *mec-4::gfp* and *myo-3::gfp* reporter genes is slower in Elongator mutants than in wild type. The effect of Elongator on translation is also consistent with the synthetic effects we observe in *elpc-1*; *tuc-1* and *elpc-3*; *tuc-1* double mutants. The reduction in accumulation of β-galactosidase activity in *elpc-1* or *elpc-3* single mutants (in which the mcm^5^ side chain of mcm^5^s^2^U containing tRNAs is absent) is similar to that seen in *tuc-1* single mutants (in which the s^2^ side chain is absent). In the double mutants (in which both the 2′and 5′modifications are lost) the efficiency of translation is further reduced. An explanation for the reduced efficiency of translation in *C. elegans* worms lacking *elpc-1* or *elpc-3* activity is that the modifications of uridine residues at the wobble position aid codon-anticodon interactions [7]–[10]. Experiments *in vivo* with *S. cereverisiae*, suggest that the primary function of the mcm^5^U, ncm^5^U and mcm^5^s^2^U nucleosides is to improve binding to A- and G- ending codons, decoded by tRNAs containing these modified nucleosides [14]–[16]. For tRNAs normally modified at both the 2′and 5′positions, the absence of either modification (or both) did not lead to any obvious misreading of U- or C-ending codons [15],[16]. Thus, presence of modifications at wobble uridines in tRNAs appears to promote the rate of elongation during translation rather than its fidelity.

There are examples of tRNA modification mutants that show temperature sensitive (ts) phenotypes, suggesting a reduced functionality of the hypomodified tRNA at the elevated temperature [16],[62],[63]. In yeast, *elp* and *tuc1* mutations result in hypomodification of 
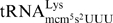
 and 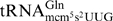 [12],[15]. In the anticodon loop, both tRNAs are rich in uridines that have a low stacking potential, and in 
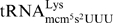
, mcm^5^ and s^2^ of U_34_ are required for a canonical anticodon loop structure [64]. Therefore, we believe that the temperature sensitive phenotype observed in *elpc* and *tuc-1* single mutants and enhanced in *elpc-1*; *tuc-1* and *elpc-3*; *tuc-1* double mutants is caused by destabilization of anticodons in hypomodified tRNAs, resulting in further weakening of codon-anticodon interactions.

### *elpc-1* and *elpc-3* Function in the Nervous System

The higher levels of expression of the *elpc::gfp* reporters within the nervous system of *C. elegans* is consistent with the finding that the most severe defects of *elpc-1* or *elpc-3* single mutants are observed in nervous system. It is interesting to note that a strong expression of Elongator subunits was also observed in the nervous system of mice [27]. A possible explanation for the greater requirement for Elongator in neurons is that neurons have markedly higher rates of protein synthesis than most other cell types [41],[65],[66]. It is also striking that in both *C. elegans* and mice, expression within the nervous system is not uniform. Perhaps different neurons have different rates of translation.

In *C. elegans* and other metazoans, neuronal function is dependent upon the ability to synthesize and secrete neurotransmitters and neuropeptides. In *elpc-1* and *elpc-3* mutants, the production of ANF::GFP neuropeptide is reduced at the posttranscriptional level. Thus Elongator mutations might cause the neurological defects by impairing the translation of neuropeptides. In addition, our findings that *elpc-1* and *elpc-3* mutants appear to have reduced levels of acetylcholine in the synaptic cleft suggest that Elongator is required for the production or secretion of neurotransmitter. Since *elpc-1::gfp* and *elpc-3::gfp* are expressed in a set of chemosensory neurons, the salt chemotaxis learning defect displayed by Elongator mutant worms is likely to be a consequence of inefficient communication among various neurons due to low production of neurotransmitters or neuropeptides. It is interesting to note that mutations in the human *ELP1* gene, also called *IKBKAP*, cause the neurodegenerative disease, Familial Dysautonomia (FD) [28],[29]. Furthermore, association studies in humans have revealed that variants at the *ELP3* locus confer increased risk for the neurodegenerative disorder Amyotrophic Lateral Sclerosis (ALS) [67]. Neuronal defects are also observed in Drosophila, Zebrafish and mouse with reduced function of *ELP3* [27],[67].

Conflicting reports exist concerning the origin of the defects caused in human cells by a reduction in hELP1/IKAP levels [25],[26],[52],[68],[69]. Recently, in mice Elongator was suggested to catalyze α-tubulin acetylation [27]. However, our observations that acetylation is not obviously abnormal in *C. elegans elpc-1* or *elpc-3* mutants suggest that the neuronal defects observed in Elongator mutants in the worm are not caused by a failure to acetylate α-tubulin.

In contrast to the *elpc-1* and *elpc-3* mutants, *tuc-1* mutants do not display defects in either the salt learning assay or in secretion of ANF::GFP. In yeast, the growth defects of Elongator mutants are more pronounced than those of the *tuc* mutants [15],[16]. One possible explanation for these differences might be that the absence of the s^2^ modification has less effect on codon-anticodon interactions than the absence of ncm^5^- and mcm^5^-groups. Alternatively, the effects on salt learning might be caused by reduced expression of a protein encoded by an mRNA rich in codons decoded by tRNAs harboring solely the 5′modification.

### Synergistic Effects with *tuc-1* Indicate Roles for ELPC-1 and ELPC-3 Outside of the Nervous System

Previous studies on *ELP1* and *ELP3* function in vertebrates have focused on their roles in neurons. While we have shown that ELPC-1 and ELPC-3 are important for nervous system function in the worm, our results clearly demonstrate that they also act in non-neural tissues. Although their expression is far from ubiquitous, the expression of ELPC::GFP reporters is clearly not restricted to neurons. More importantly, the defects in temperature-shifted *elpc-1*; *tuc-1* and *elpc-3*; *tuc-1* double mutants indicate that Elongator is also involved in embryogenesis and vulval development. The phenotypes observed suggest that tRNA modification is a mechanism by which the efficiency of translation is modulated during metazoan development.

### Concluding remarks

Our observations suggest that Elongator acts in neurological and developmental processes in *C. elegans* by modulating translation. An important task in the future is to identify the mRNAs whose translation is dependent on Elongator activity. Identification of these mRNAs might help in the understanding of the molecular mechanisms of Elongator-related human diseases.

## Materials and Methods

### Nomenclature of Genes and Neurons

The names of genes in the text have been given according to existing nomenclature rules for *S. cerevisiae*, *C. elegans* and humans. The yeast *ELP1* gene encodes a protein, Elp1p; the equivalent gene in *C. elegans*, *elpc-1* encodes ELPC-1; in humans, *IKBKAP*/*hELP1* encodes IKAP/hELP1. The respective mutant alleles are *elp1* (*S. cerevisiae*) and *elpc-1*(*tm2149*) (*C. elegans*). Neurons in *C. elegans* have three-letter names *e. g.*, ASE. These names are not acronyms or abbreviations.

### Strains

*C. elegans* worms were cultured and handled as described previously [70]. All strains were maintained at 20°C unless likewise indicated. All are derived from the wild-type strain, Bristol N2 [70]. For routine propagation, worms were maintained on nematode growth medium (NGM) plates [70]. The following mutations were used in this study. Linkage group (*LG*) *I*, *tom-1*(*ok285*) [71]–[73], *aex-6*(*sa24*) [50], *lev-11*(*x11*) [74]; *elpc-1*(*tm2149*), *LG III*, *mec-12*(*e1605*), *mec-12*(*e1607*) [75], *LG IV*, *tuc-1*(*tm1297*) [39]; *LG V*, *elpc-3*(*tm3120*). The transgenes used were *ubIn5*[*hsp16::lacZ*] [76], *oxIs180*[P*_aex-3_::ANF::gfp*] [47], *svEx557*[P*_elpc-1_::elpc-1::gfp*], *zdIs5 I*[*mec-4::gfp lin-15*(+)] [77], *svEx666*[*lin-25::HA myo-3::gfp*], *svEx806*[*elpc-1::gfp Punc-122::gfp*], *svEx808*[*elpc-1::gfp Punc-122::gfp*], *adEx1262*[*gcy-5::gfp lin-15*(+)] [78]. The *elpc-1*, *elpc-3* and *tuc-1* deletion mutants were backcrossed eight times with wild-type N2 before use.

### Plasmid Construction

To generate the *elpc-1::gfp* fusion, the entire *elpc-1* coding region together with 435 base pairs of DNA upstream of the start ATG was amplified by using primers 5′-AAAAGCATGCTCCGGTACGGTATGTGGC-3′ and 5′-AAAACTGCAGTGGGAAAACTGAAG CAAATGAA-3′. The PCR product was subcloned into pPD95.77 GFP expression vector between PstI and SphI sites.

### DIC and Immunofluorescence Microscopy of Nematodes

A Leica DMRB microscope equipped with both Nomarski differential interference contrast and epifluorescence optics was used to view worms at high magnification. Images were captured with a Deltpix CCD camera and software (Deltapix, Copenhagen). Confocal microscopy was performed on a Leica TCS SP2 confocal microscope. Confocal images were captured using Leica confocal software.

### tRNA Isolation and HPLC Analysis

Techniques described by Gaur *et al.* (2007) were used with minor modifications to isolate and analyze tRNA from *C. elegans* worms. For each strain, worms from twenty 9 cm culture plates containing mixed-stage populations of worms were used. After extensive washing with M9 buffer, the worm pellets were frozen in the liquid nitrogen and then thawed in the presence of 0.5 volumes of TRIzol (Invitrogen). A tissue-grinder (Kontes) was used to break open the worms. After extraction of the lysate with chloroform, followed by addition of isopropanol, total RNA was sedimented by centrifugation. tRNA was separated from other types of RNA by using methods described previously [79]. Purified tRNA was digested with Nuclease P1 for 16 h at 37°C and then treated with bacterial alkaline phosphatase for 2 h at 37°. The hydrolysate was analyzed by high pressure liquid chromatography with a Develosil C-30 reverse-phase column as described [79]. ncm^5^U, mcm^5^U, mcm^5^s^2^U and mcm^5^U_m_ have all been found on wobble uridines in *S. cerevisiae* tRNA. We did not examine *C. elegans* tRNA for the presence of ncm^5^U_m_ because P1 and BAP cannot digest the dinucleotide ncm^5^U_m_pX to nucleosides [80].

### RNA Isolation and Quantitative RT–PCR

Total RNA was extracted with the aid of a BIO-RAD Aurum total RNA mini kit according to the instruction manual. Real-time PCR was carried out in 25 µl reaction mixes. iScript one-step RT-PCR kit with SYBR green (BIO-RAD) and the iCycler iQ Real-Time PCR Detection System (BIO-RAD) were used. The data were normalized to *tbb-2* and *ubc-2* mRNA levels. Six independent assays were performed for each strain analyzed.

### β-Galactosidase Assay

For each strain analyzed, one 6-cm plate containing a population of well-fed worms was subjected to a 2 h heat shock at 33°. The worms were washed from the plate with M9 salt solution, sedimented, washed once in M9 and then once in breaking buffer (100 mM Tris-HCl, 1 mM DTT, 20% glycerol). After resuspension in 250 µl of breaking buffer containing Roche protease inhibitor cocktail, the worms were broken open by sonication. Five 2 sec pulses at maximum effect were used. The extracts were transferred to microcentrifuge tubes and worm debris was sedimented by centrifugation at 13,000 rpm for 15 min. β-galactosidase activity in the cleared extracts was measured using standard protocols [81].

### Fluorescence Recovery after Photobleaching (FRAP)

The assay was performed as described in detail by Kourtis and Tavernarakis [41]. Worms carrying the *gcy-5::gfp*, *mec-4::gfp* or *myo-3::gfp* reporters were mounted on the agarose pad in the presence of levamisol and photobleached with light from an HBO 103W/2 mercury lamp (OSRAM). A 63× objective was used for photobleaching *gcy-5::gfp* and *mec-4::gfp* strains, a 20× objective for *myo-3::gfp* strains.

### Chemotaxis Assay

Salt chemotaxis assays were performed as described by Ward [82] and Bargmann and Horvitz [44]. All the assays were carried out at room temperature (*ca.* 21.5°C) on 9 cm agar plates containing 5 mM KH_2_PO_4_ pH 6.0, 1 mM CaCl_2_, 1 mM MgSO_4_ and 2% agar. N2, *elpc-1(tm2149)*, *elpc-3(tm3120)* and *tuc-1(1297)* strains were maintained at 25°C for at least three generations prior to being assayed. The salt gradient with a peak 0.5 cm from one edge of the plate was formed overnight by placing a block of agar measuring approximately 5 mm in each dimension and containing 100 mM NaCl, 5 mM KH_2_PO_4_ pH 6.0, 1 mM CaCl_2_, 1 mM MgSO_4_ and 2% agar. In each single test, 80–100 young adult worms were washed three times in 5 mM KH_2_PO_4_ pH 6.0, 1 mM CaCl_2_, 1 mM MgSO_4_ and then placed in the center of the assay plates. Before the worms were placed on the assay plate, 1 µl of 0.5 M sodium azide was spotted both at the salt gradient peak and at the opposite side of the plate to capture the worms moving to those areas. The numbers of worms at different positions on the plate were counted every 10 min after the start of the assay. The formula 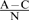 was used to calculate the chemotaxis index. In this equation, A was the number of worms at the attractant area, C the number of worms at the control spot, and N the total number of worms placed on the plates. Each experiment was repeated at least 4 times. For chemotaxis assays with isoamyl alcohol, the odorant was dropped on the assay plate immediately prior to the addition of worms to the plate.

### Salt Learning Assay

The assay was performed as described [43], with minor modifications. For each assay, adult worms were washed off the culture plates with chemotaxis washing buffer (5 mM KH_2_PO_4_ pH 6.0, 1 mM CaCl_2_, 1 mM MgSO_4_) and then washed three times in the same buffer. For the naive condition, worms were washed and then assayed immediately without further incubation. The other worms were conditioned respectively on nematode growth medium (NGM) plates containing 100 mM NaCl, or on NaCl-free NGM plates for 4 hours. After conditioning, worms were collected again and placed on the assay plates. After 30 min, the numbers of worms in the NaCl spot (A) and the control region (C) were counted. The index was calculated using the formula, 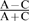.

### Western Blot

ANF::GFP levels were measured by western blotting using an anti-GFP antibody (Clontech, JL-8). 50 L4 larvae of each genotype were collected, boiled in SDS sample buffer for 5 min and loaded onto a 10% SDS PAGE. Quantification of imaging pixel intensity was performed by NIH image J. To measure acetylated α-tubulin levels by western blot, protein was extracted from young adult worms. To avoid protein degradation, worms were suspended in ice-cold extraction buffer containing proteinase inhibitors and rapidly frozen in liquid nitrogen. The frozen pellets were ground to a powder in a mortar. 20 µg protein was loaded on the gel in each lane. The dilution of anti-lys40-acetylated-α-tubulin antibody (abcam, 6-11B-1) was 1∶1000, and of anti-α-tubulin antibody (Sigma, B-5-1-2) was 1∶2000.

### Measurment of Aldicarb and Levamisol Sensitivity

The assays were performed as described by Mahoney *et al.* [49]. 25–30 worms were used for each genotype. The assay was performed blind in triplicate at room temperature (*ca.* 21.5°C). The worms were cultivated at 25°C prior to being assayed.

### Quantification of ANF::GFP Fluorescence in Coelomocytes

The assay was performed as described by Speese *et al.* [47]. Fluorescence confocal micrographs were made of coelomocytes. The intensity of GFP fluorescence in captured images in grey scale was measured with the aid of the NIH ImageJ software.

## Supporting Information

Figure S1 ***C. elegans elpc-3*** **is required for mcm^5^ and ncm^5^ side chain formation at wobble uridines.** (A) The diagram shows the genomic structures of *elpc-3* and ZK863.4, which is suggested to be in the same operon. The exons and introns are depicted as boxes and lines respectively. At top, the line underneath represents the location of the deletion in *elp-3(tm3120)*. Below, a representation of predicted motifs in the ELPC-3 protein: the Radical S-adenosyl methionine (Radical-SAM) [31], and histone acetyltransferase (HAT) domains [83]. The region deleted in *elpc-3(tm3120)* is indicated by a line beneath. (B–G) Chromatograms of total tRNA isolated from wild-type and *elpc-3(tm3120)* worms analyzed by HPLC. Wild-type (N2) profiles are shown in the left panels; *elpc-3(tm3120)* profiles are shown in the right panels. Chromatograms were monitored at 254 nm, unless otherwise stated. (B,C) The parts of chromatograms between retention times 46 and 51.5 min are displayed. The arrow in C indicates the expected retention time of mcm^5^s^2^U. (D,E) The parts of the chromatograms between retention times 11 and 18 min are displayed. The arrow in E indicates the expected retention time of ncm^5^U. (F,G) The parts of the chromatograms between retention times 31 and 37 min are displayed. The arrow in F indicates the expected retention time of s^2^U. Chromatograms were monitored at 314 nm.
(0.55 MB TIF)

Figure S2 ***tuc-1*** **in** ***C. elegans*** **is required for 2-thio wobble uridine tRNA modification.** (A) The schematic structure of *tuc-1*. Exons and introns are represented by boxes and lines, respectively. The line underneath indicates the region deleted in *tuc-1(tm1297)*. (B–E) Chromatograms showing total tRNA isolated from wild-type (N2) and *tuc-1(tm1297)* worms analyzed by HPLC. N2 profiles are shown in the left panels; *tuc-1(tm1297)* profiles are shown in the right panels. Chromatograms were monitored at 254 nm. (B,C) The parts of the chromatograms between retention times 46.2 and 51.6 min are displayed. The arrow in C indicates the expected retention time of mcm^5^s^2^U. (D,E) The parts of the chromatograms between retention times 35 and 42 min are displayed. The arrow in D indicates the expected retention time of mcm^5^U.
(0.43 MB TIF)

Figure S3 **Formation of ncm^5^, mcm^5^ and s^2^ side chains is abolished in** ***elpc-1*** **;** ***tuc-1*** **double mutants.** (A–F) Total tRNA isolated from wild type, *elpc-1(tm2149)* or *elpc-1(tm2149)*; *tuc-1(tm1297)* worms was analyzed by HPLC. Wild-type (N2) and *elpc-1(tm2149)* profiles are shown in left panels. *elpc-1(tm2149)*; *tuc-1(tm1297)* profiles are in right panels. Chromatograms were monitored at 254 nm, unless otherwise stated. (A,B) The parts of chromatograms between retention times 48.5 and 53.5 min are displayed. The arrow in the right panel indicates the expected retention time of mcm^5^s^2^U. (C,D) The parts of the chromatograms between retention times 11 and 18 min are displayed. The arrow in the right panel indicates the expected retention time of ncm^5^U. (E,F) The parts of the chromatograms between retention times 33 and 39 min are displayed. The arrow in the right panel indicates the expected retention time of s^2^U. Chromatograms were monitored at 314 nm.
(0.45 MB TIF)

Figure S4 **The** ***elp-1(tm2149)*** **and** ***elpc-3(tm3120)*** **mutants are defective in fluorescence recovery after photobleaching.** Quantification of fluorescence signals in worms carrying *gcy-5::gfp* (A) or *myo-3::gfp* (B) reporters. The pixel intensities in wild type, *elpc-1*, *elpc-3* and *tuc-1* backgrounds before photobleaching, after photobleaching, and after 5 hours recovery are shown. In ‘*gcy-5::gfp*+CHX’, fluorescence recovery was measured in the presence of cycloheximide (CHX). The number of worms examined of each strain is denoted under the graph. Error bars represent standard deviations.
(0.67 MB TIF)

Figure S5 **Neuronal morphology in** ***elpc-1*** **,** ***elpc-3*** **and** ***tuc-1*** **mutant worms is normal.** (A, C, E, G, I, K) Micrographs of hermaphrodite worms fed with DiI viewed with Nomarski DIC optics. The arrows denote three amphid neurons, ASI, ADL and ASK. (B, D, F, H, J, L) The same worms viewed with fluorescence optics. Note that DiI efficiently labels the neurons in the mutant worms, indicating that the outgrowth of the neuronal processes was normal.
(5.94 MB TIF)

Figure S6 **The salt chemotaxis learning defect of** ***elpc-1(tm2149)*** **is rescued by an** ***elpc-1::gfp*** **construct.** Worms were synchronized and raised at 25°C to the young adult stage. The chemotaxis index after 30 min of assay is displayed. The assay was repeated four times. Error bars denote standard deviations. Two asterisks indicate a significant difference between *elpc-1* and *elpc-1; elpc-1::gfp* (**p<0.001 by student's t test).
(0.20 MB TIF)

Figure S7 ***elpc-1*** **,** ***elpc-3*** **, and** ***tuc-1*** **chemotax to both NaCl and isoamylalcohol.** (A, B) Chemotaxis to NaCl (A) and isoamyl alcohol (B) is shown. The chemotaxis indices were plotted against time for four different genotypes. For each genotype, 80–100 young adult worms that had been raised at 25°C were placed on a plate equidistant from the attractant and a control spot. The numbers of worms at the NaCl (or isoamylalcohol) and the control spots were counted every 10 minutes for 1 hour. Each assay was repeated for 4 times.
(0.26 MB TIF)

Figure S8 **The increased aldicarb resistance of the** ***elpc-1(tm2149)*** **mutant is complemented by** ***elpc-1::gfp*** **.** The proportion of worms still able to move is plotted against time. 25–30 worms were used for each genotype. The assay was performed blind in triplicate at room temperature (*ca.* 21.5°C). The worms were cultivated at 25°C prior to being assayed.
(0.22 MB TIF)

Figure S9 **Acetylated α-tubulin levels are not decreased in Elongator mutants.** (A) Western blot of whole animal lysates for wild type (N2), *elpc-1*, *elpc-3*, *mec-12(e1605)* and *mec-12(e1607)*. Both acetylated α-tubulin and α-tubulin migrated just above 50 KDa. Top, blotted with anti-lys40-acetylated-α-tubulin antibody at a dilution of 1∶1000. Bottom, blotted with anti-α-tubulin antibody at a dilution of 1∶2000. Lys40 acetylated α-tubulin signals were normalized to that of α-tubulin, and the amount of lys40 acetylated α-tubulin was expressed relative to the corresponding value in the wild type strain, which was set to 1. NA, not applicable. (B) *mec-12(e1607)* worms are not resistant to aldicarb. The proportion of worms still able to move is plotted against time. 25–30 worms were used for each genotype. The assay was performed blind in triplicate at room temperature (*ca.* 21.5°C). The worms were cultivated at 25°C prior to being assayed.
(0.66 MB TIF)

Figure S10 **Defects Seen in Temperature-shifted** ***elpc-3*** **;** ***tuc-1*** **Double Mutants.** Micrographs of eggs and larvae viewed with Nomarski DIC optics. (A–D) Embryos arrested prior to (A), during (B,C) or after (D) morphogenesis. (E,F,G,H) Parts of the germline in young adult hermaphrodites. The arrows in E and F indicate oocytes. Note that those in the *elpc-3; tuc-1* worm have not matured. The arrows in G and H denote sperm. Those in the *elpc-3*; *tuc-1* worm have grossly abnormal morphology. (I,J) The descendants of P5.p, P6.p and P7.p during the L4 stage. The arrows in I denote the descendants of P5.p, P6.p and P7.p. In the animal shown, these three cells adopted the 3° cell fate and divided just once. In wild-type worms, P6.p adopts the 1° cell fate whereas P5.p and P7.p adopt the 2° fate. The 1° and 2° fates involve three rounds of cell division; the descendants of P5.p, P6.p and P7.p together form a tube through which the eggs are laid. The arrow in J denotes the tube as it is forming. Scale bars denote 10 microns.
(2.42 MB TIF)

Figure S11 **ELPC-1::GFP is expressed during vulval cell fate specification.** Micrographs of an L3 hermaphrodite worm of the genotype *elpc-1(tm2149)*; *svEx557*[P*_elpc-1_::elpc-1::gfp*] viewed with either Nomarski differential contrast (DIC) (A) or fluorescence (B) optics. The arrows denote the six descendants of P5.p, P6.p and P7.p.
(1.58 MB TIF)
