# Supplementary figures and images for: Defects in tRNA Modification Associated with Neurological and Developmental Dysfunctions in Caenorhabditis elegans Elongator Mutants
Source: PLoS Genet. 2009 Jul 10;5(7):e1000561. doi: 10.1371/journal.pgen.1000561 (PMC2702823; doi:10.1371/journal.pgen.1000561)

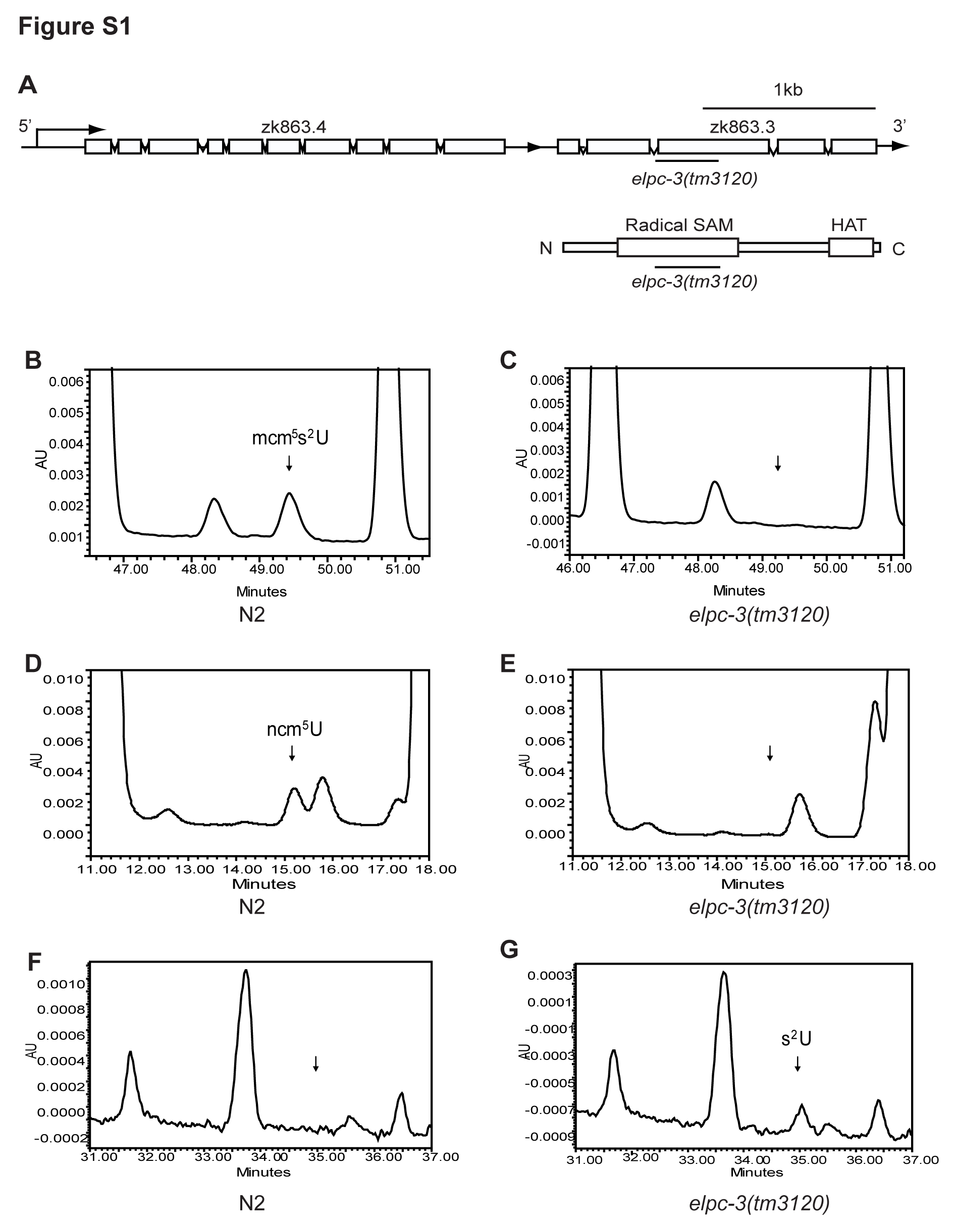

Supplement: Figure S1 — C. elegans elpc-3 is required for mcm5 and ncm5 side chain formation at wobble uridines. (A) The diagram shows the genomic structures of elpc-3 and ZK863.4, which is suggested to be in the same operon. The exons and introns are depicted as boxes and lines respectively. At top, the line underneath represents the location of the deletion in elp-3(tm3120). Below, a representation of predicted motifs in the ELPC-3 protein: the Radical S-adenosyl methionine (Radical-SAM) [31], and histone acetyltransferase (HAT) domains [83]. The region deleted in elpc-3(tm3120) is indicated by a line beneath. (B–G) Chromatograms of total tRNA isolated from wild-type and elpc-3(tm3120) worms analyzed by HPLC. Wild-type (N2) profiles are shown in the left panels; elpc-3(tm3120) profiles are shown in the right panels. Chromatograms were monitored at 254 nm, unless otherwise stated. (B,C) The parts of chromatograms between retention times 46 and 51.5 min are displayed. The arrow in C indicates the expected retention time of mcm5s2U. (D,E) The parts of the chromatograms between retention times 11 and 18 min are displayed. The arrow in E indicates the expected retention time of ncm5U. (F,G) The parts of the chromatograms between retention times 31 and 37 min are displayed. The arrow in F indicates the expected retention time of s2U. Chromatograms were monitored at 314 nm. (0.55 MB TIF) [file pgen.1000561.s001.tif]

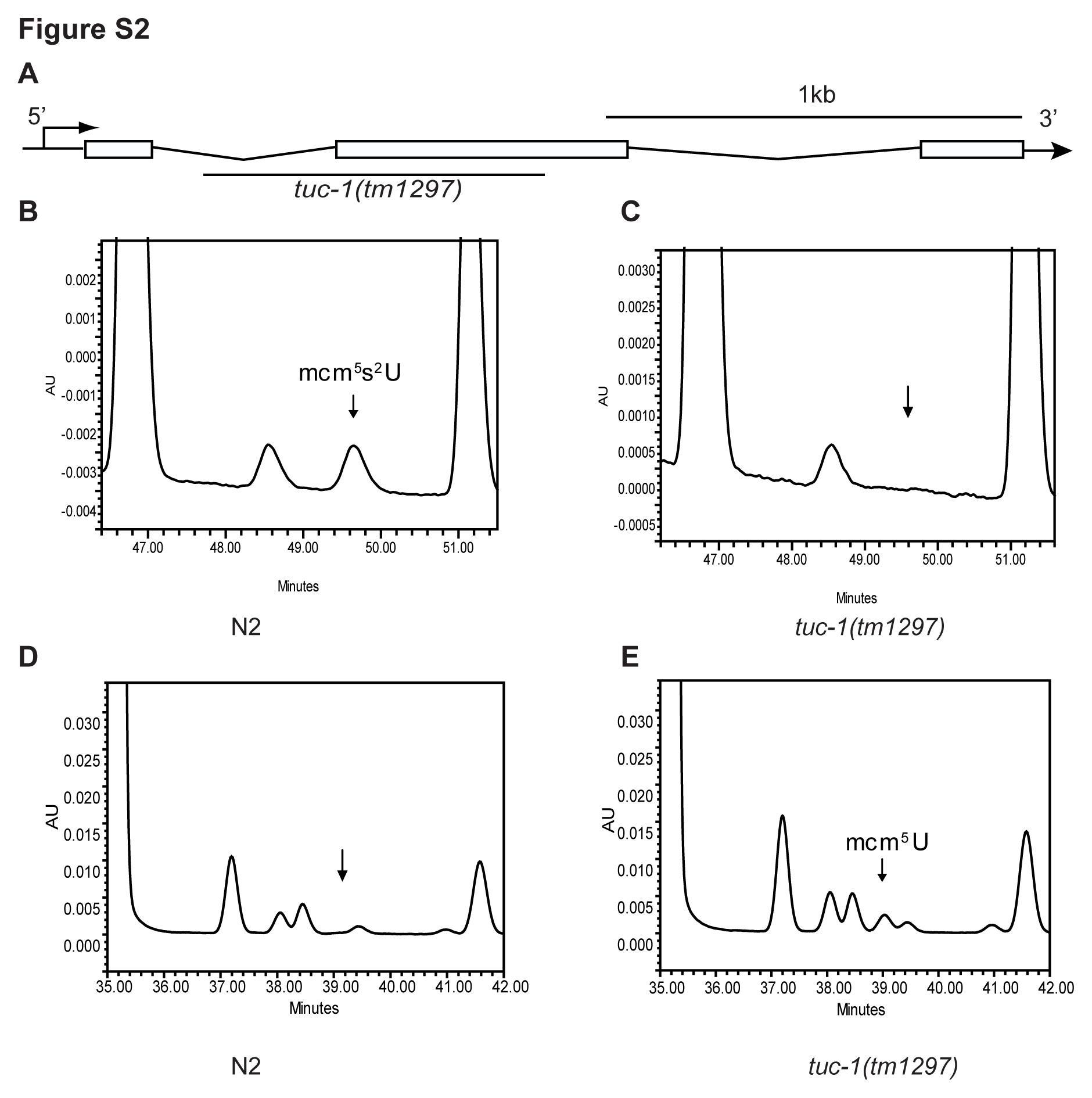

Supplement: Figure S2 — tuc-1 in C. elegans is required for 2-thio wobble uridine tRNA modification. (A) The schematic structure of tuc-1. Exons and introns are represented by boxes and lines, respectively. The line underneath indicates the region deleted in tuc-1(tm1297). (B–E) Chromatograms showing total tRNA isolated from wild-type (N2) and tuc-1(tm1297) worms analyzed by HPLC. N2 profiles are shown in the left panels; tuc-1(tm1297) profiles are shown in the right panels. Chromatograms were monitored at 254 nm. (B,C) The parts of the chromatograms between retention times 46.2 and 51.6 min are displayed. The arrow in C indicates the expected retention time of mcm5s2U. (D,E) The parts of the chromatograms between retention times 35 and 42 min are displayed. The arrow in D indicates the expected retention time of mcm5U. (0.43 MB TIF) [file pgen.1000561.s002.tif]

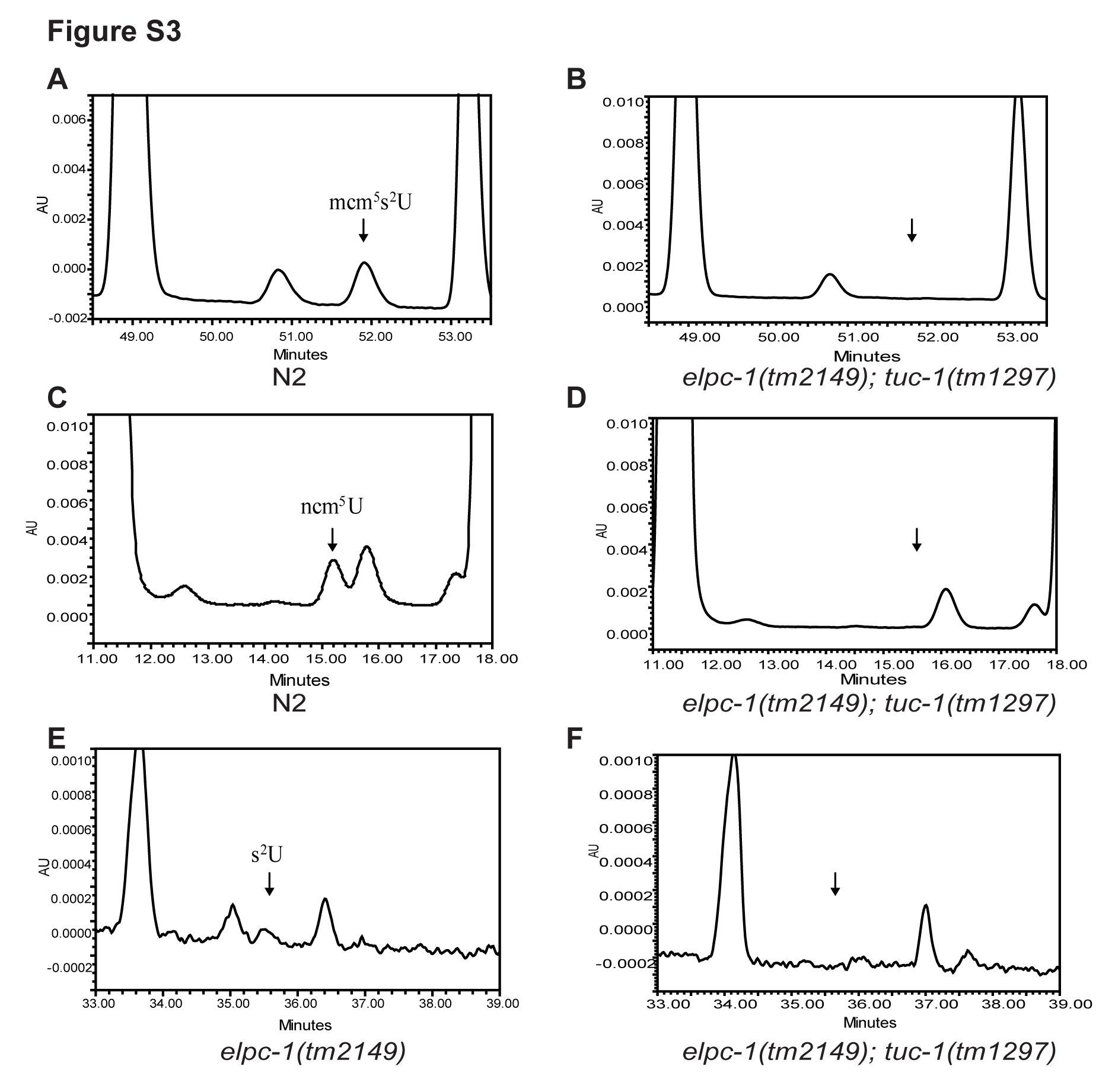

Supplement: Figure S3 — Formation of ncm5, mcm5 and s2 side chains is abolished in elpc-1 ; tuc-1 double mutants. (A–F) Total tRNA isolated from wild type, elpc-1(tm2149) or elpc-1(tm2149); tuc-1(tm1297) worms was analyzed by HPLC. Wild-type (N2) and elpc-1(tm2149) profiles are shown in left panels. elpc-1(tm2149); tuc-1(tm1297) profiles are in right panels. Chromatograms were monitored at 254 nm, unless otherwise stated. (A,B) The parts of chromatograms between retention times 48.5 and 53.5 min are displayed. The arrow in the right panel indicates the expected retention time of mcm5s2U. (C,D) The parts of the chromatograms between retention times 11 and 18 min are displayed. The arrow in the right panel indicates the expected retention time of ncm5U. (E,F) The parts of the chromatograms between retention times 33 and 39 min are displayed. The arrow in the right panel indicates the expected retention time of s2U. Chromatograms were monitored at 314 nm. (0.45 MB TIF) [file pgen.1000561.s003.tif]

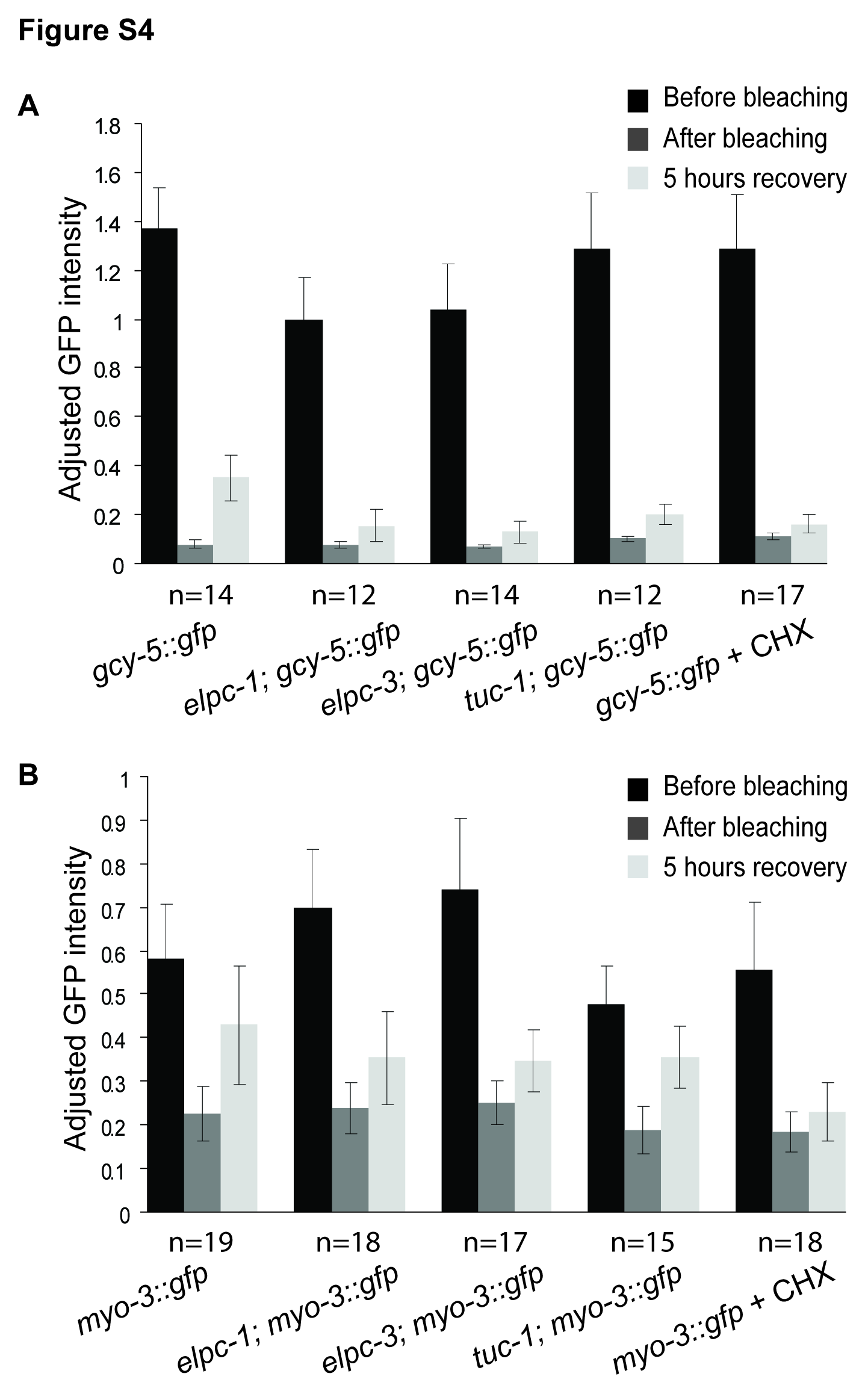

Supplement: Figure S4 — The elp-1(tm2149) and elpc-3(tm3120) mutants are defective in fluorescence recovery after photobleaching. Quantification of fluorescence signals in worms carrying gcy-5::gfp (A) or myo-3::gfp (B) reporters. The pixel intensities in wild type, elpc-1, elpc-3 and tuc-1 backgrounds before photobleaching, after photobleaching, and after 5 hours recovery are shown. In ‘gcy-5::gfp+CHX’, fluorescence recovery was measured in the presence of cycloheximide (CHX). The number of worms examined of each strain is denoted under the graph. Error bars represent standard deviations. (0.67 MB TIF) [file pgen.1000561.s004.tif]

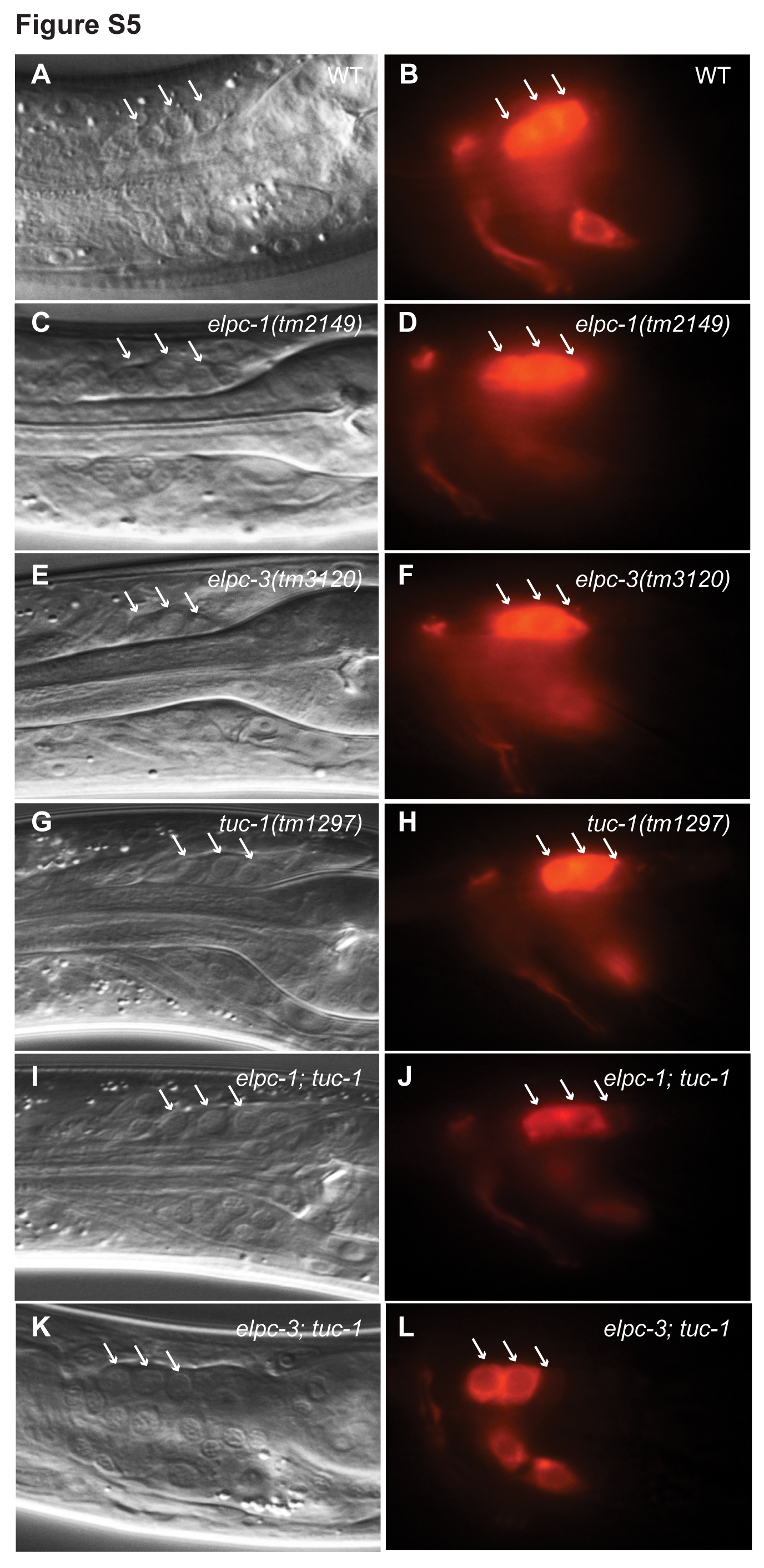

Supplement: Figure S5 — Neuronal morphology in elpc-1 , elpc-3 and tuc-1 mutant worms is normal. (A, C, E, G, I, K) Micrographs of hermaphrodite worms fed with DiI viewed with Nomarski DIC optics. The arrows denote three amphid neurons, ASI, ADL and ASK. (B, D, F, H, J, L) The same worms viewed with fluorescence optics. Note that DiI efficiently labels the neurons in the mutant worms, indicating that the outgrowth of the neuronal processes was normal. (5.94 MB TIF) [file pgen.1000561.s005.tif]

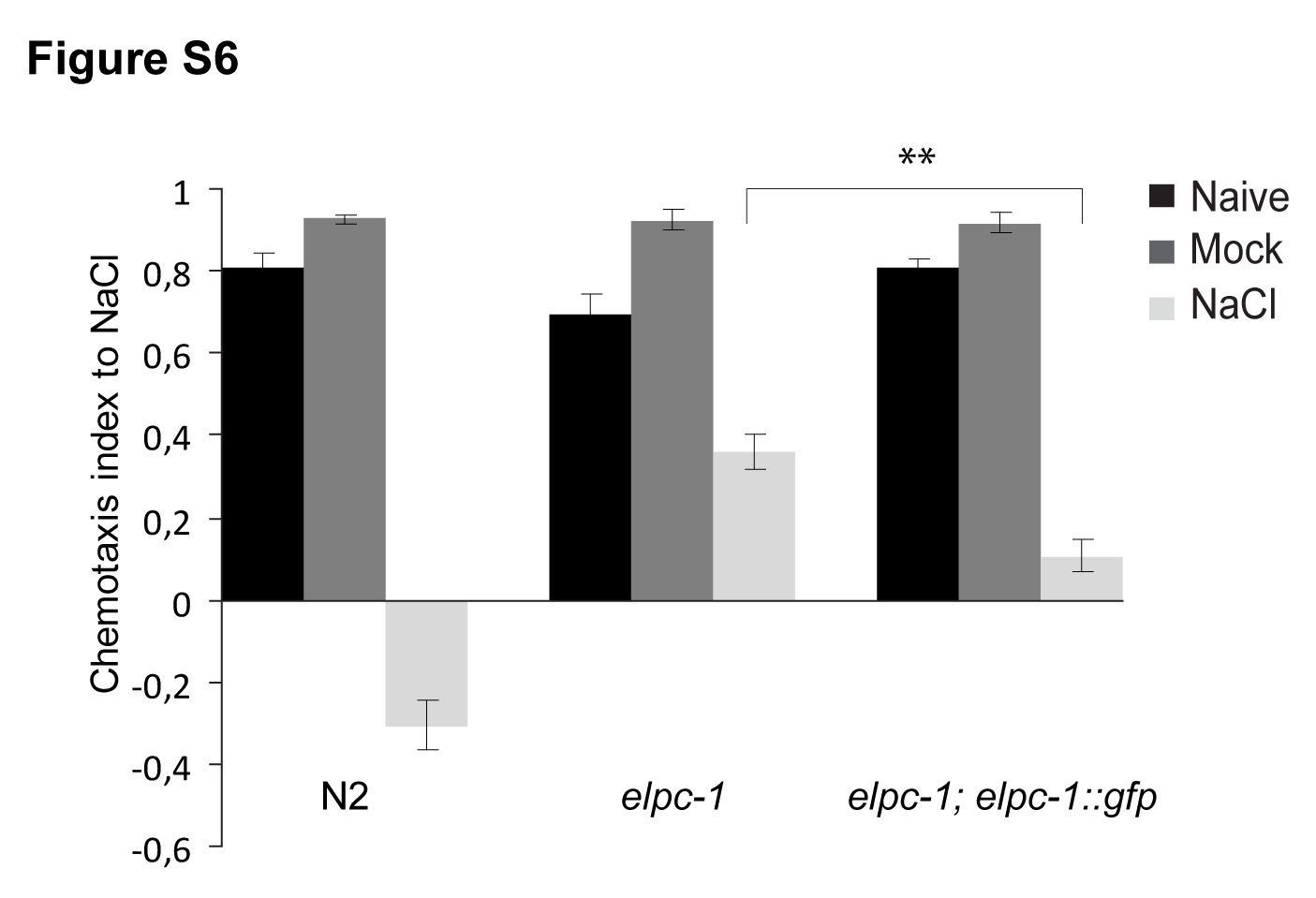

Supplement: Figure S6 — The salt chemotaxis learning defect of elpc-1(tm2149) is rescued by an elpc-1::gfp construct. Worms were synchronized and raised at 25°C to the young adult stage. The chemotaxis index after 30 min of assay is displayed. The assay was repeated four times. Error bars denote standard deviations. Two asterisks indicate a significant difference between elpc-1 and elpc-1; elpc-1::gfp (**p<0.001 by student's t test). (0.20 MB TIF) [file pgen.1000561.s006.tif]

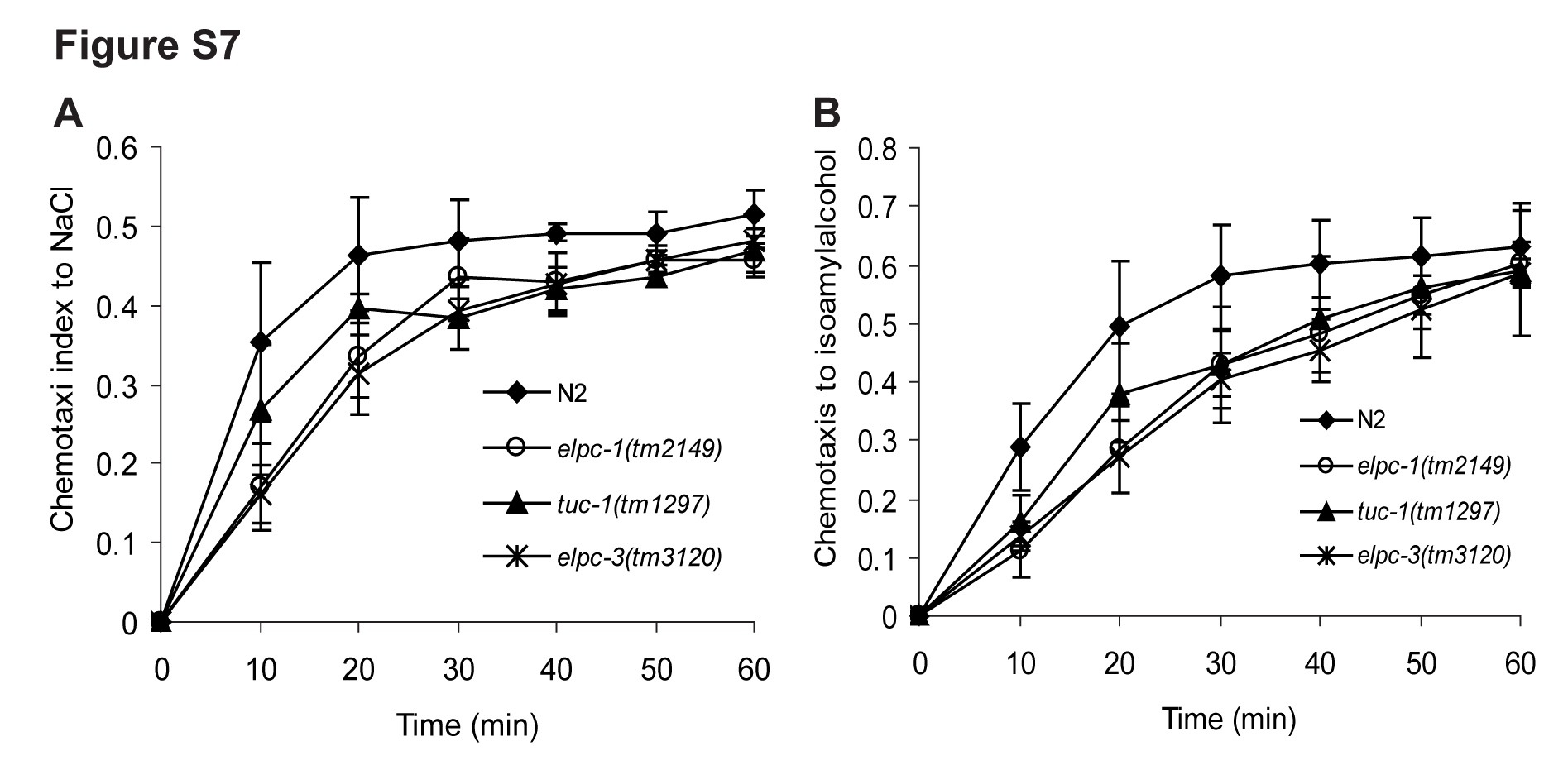

Supplement: Figure S7 — elpc-1 , elpc-3 , and tuc-1 chemotax to both NaCl and isoamylalcohol. (A, B) Chemotaxis to NaCl (A) and isoamyl alcohol (B) is shown. The chemotaxis indices were plotted against time for four different genotypes. For each genotype, 80–100 young adult worms that had been raised at 25°C were placed on a plate equidistant from the attractant and a control spot. The numbers of worms at the NaCl (or isoamylalcohol) and the control spots were counted every 10 minutes for 1 hour. Each assay was repeated for 4 times. (0.26 MB TIF) [file pgen.1000561.s007.tif]

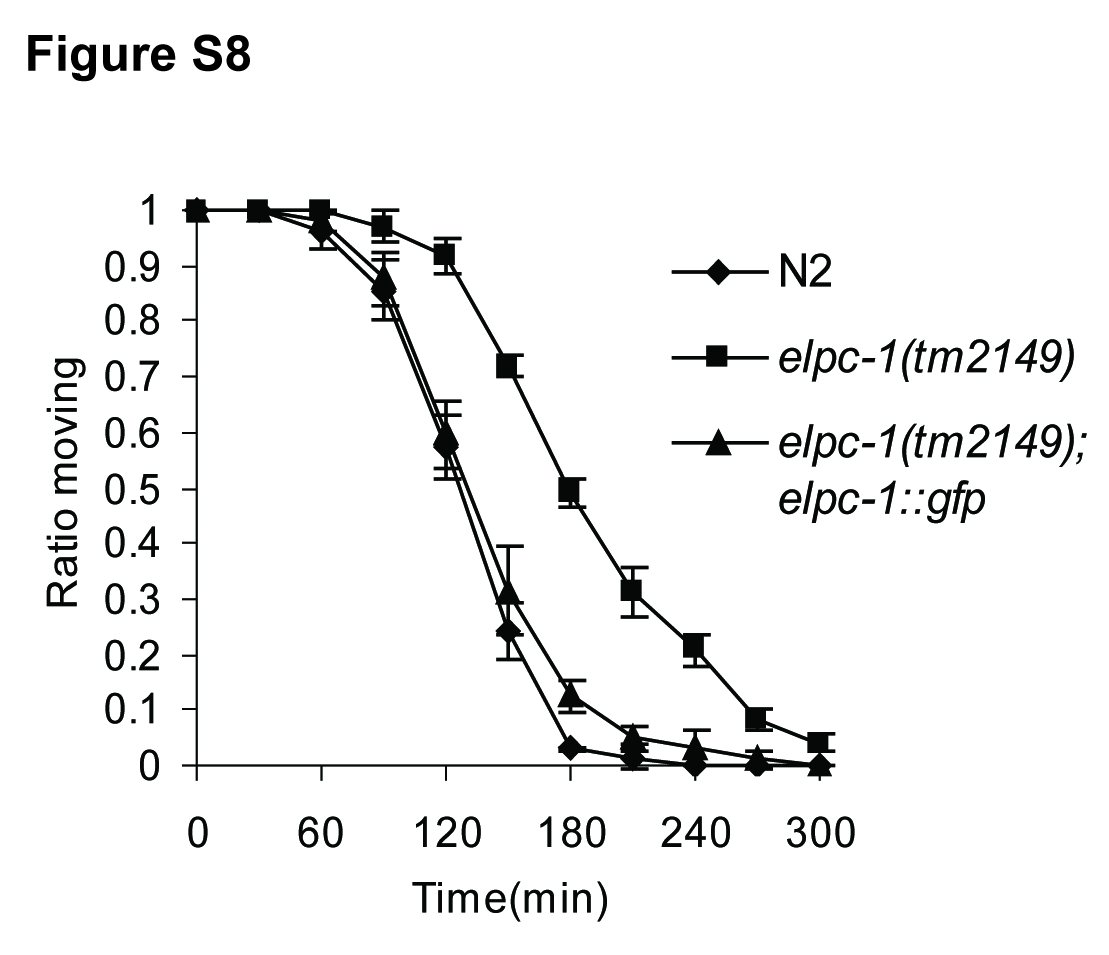

Supplement: Figure S8 — The increased aldicarb resistance of the elpc-1(tm2149) mutant is complemented by elpc-1::gfp . The proportion of worms still able to move is plotted against time. 25–30 worms were used for each genotype. The assay was performed blind in triplicate at room temperature (ca. 21.5°C). The worms were cultivated at 25°C prior to being assayed. (0.22 MB TIF) [file pgen.1000561.s008.tif]

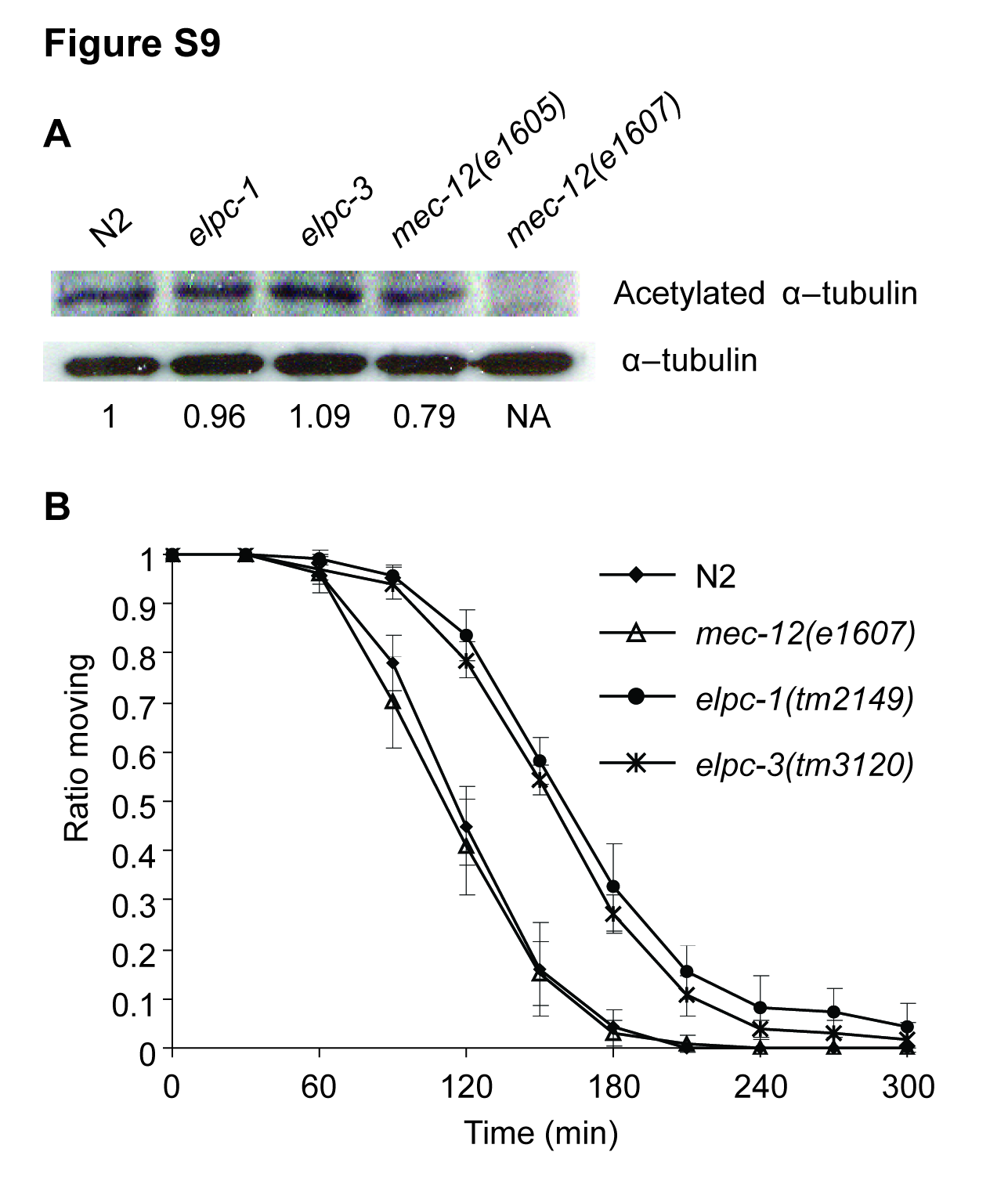

Supplement: Figure S9 — Acetylated α-tubulin levels are not decreased in Elongator mutants. (A) Western blot of whole animal lysates for wild type (N2), elpc-1, elpc-3, mec-12(e1605) and mec-12(e1607). Both acetylated α-tubulin and α-tubulin migrated just above 50 KDa. Top, blotted with anti-lys40-acetylated-α-tubulin antibody at a dilution of 1∶1000. Bottom, blotted with anti-α-tubulin antibody at a dilution of 1∶2000. Lys40 acetylated α-tubulin signals were normalized to that of α-tubulin, and the amount of lys40 acetylated α-tubulin was expressed relative to the corresponding value in the wild type strain, which was set to 1. NA, not applicable. (B) mec-12(e1607) worms are not resistant to aldicarb. The proportion of worms still able to move is plotted against time. 25–30 worms were used for each genotype. The assay was performed blind in triplicate at room temperature (ca. 21.5°C). The worms were cultivated at 25°C prior to being assayed. (0.66 MB TIF) [file pgen.1000561.s009.tif]

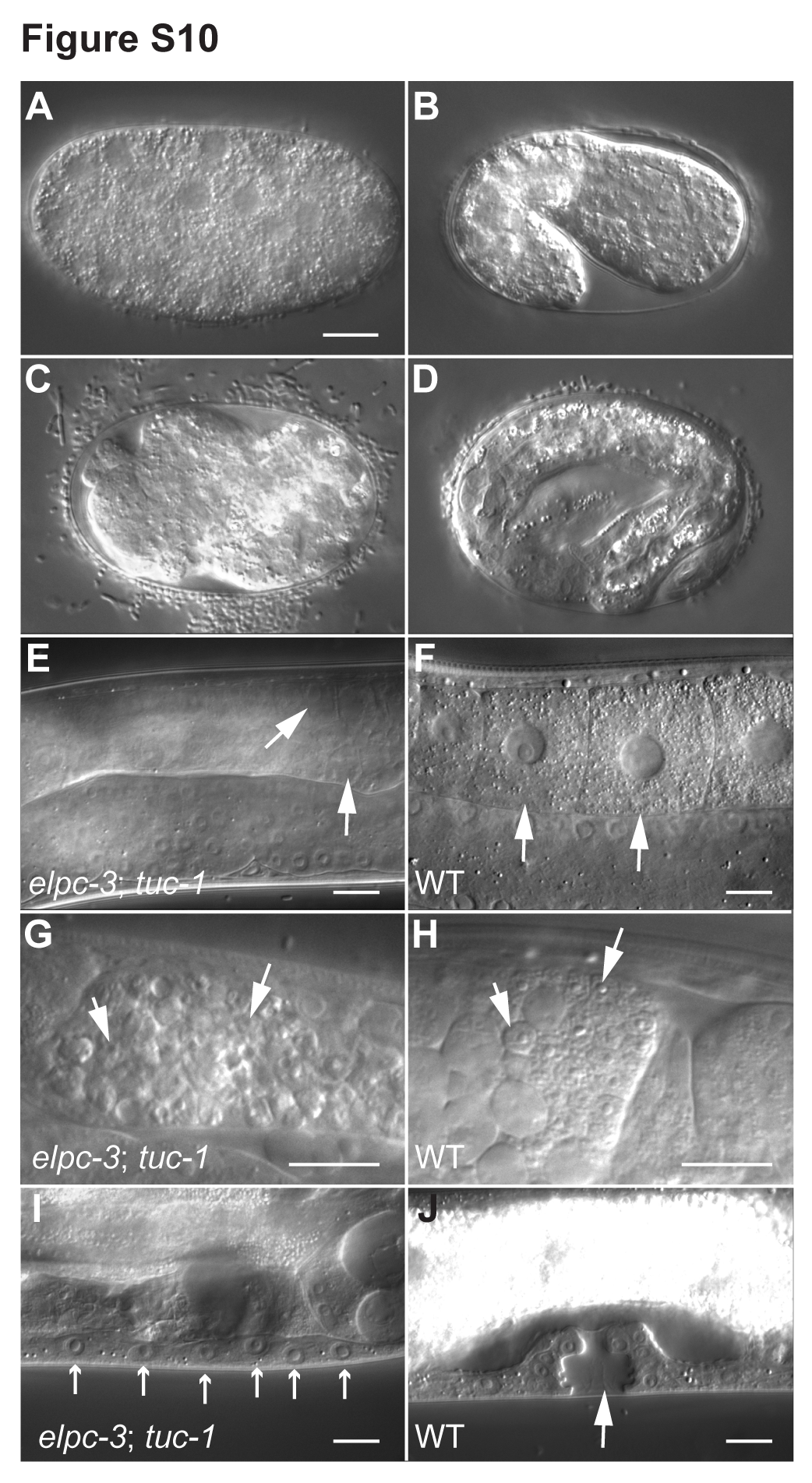

Supplement: Figure S10 — Defects Seen in Temperature-shifted elpc-3 ; tuc-1 Double Mutants. Micrographs of eggs and larvae viewed with Nomarski DIC optics. (A–D) Embryos arrested prior to (A), during (B,C) or after (D) morphogenesis. (E,F,G,H) Parts of the germline in young adult hermaphrodites. The arrows in E and F indicate oocytes. Note that those in the elpc-3; tuc-1 worm have not matured. The arrows in G and H denote sperm. Those in the elpc-3; tuc-1 worm have grossly abnormal morphology. (I,J) The descendants of P5.p, P6.p and P7.p during the L4 stage. The arrows in I denote the descendants of P5.p, P6.p and P7.p. In the animal shown, these three cells adopted the 3° cell fate and divided just once. In wild-type worms, P6.p adopts the 1° cell fate whereas P5.p and P7.p adopt the 2° fate. The 1° and 2° fates involve three rounds of cell division; the descendants of P5.p, P6.p and P7.p together form a tube through which the eggs are laid. The arrow in J denotes the tube as it is forming. Scale bars denote 10 microns. (2.42 MB TIF) [file pgen.1000561.s010.tif]

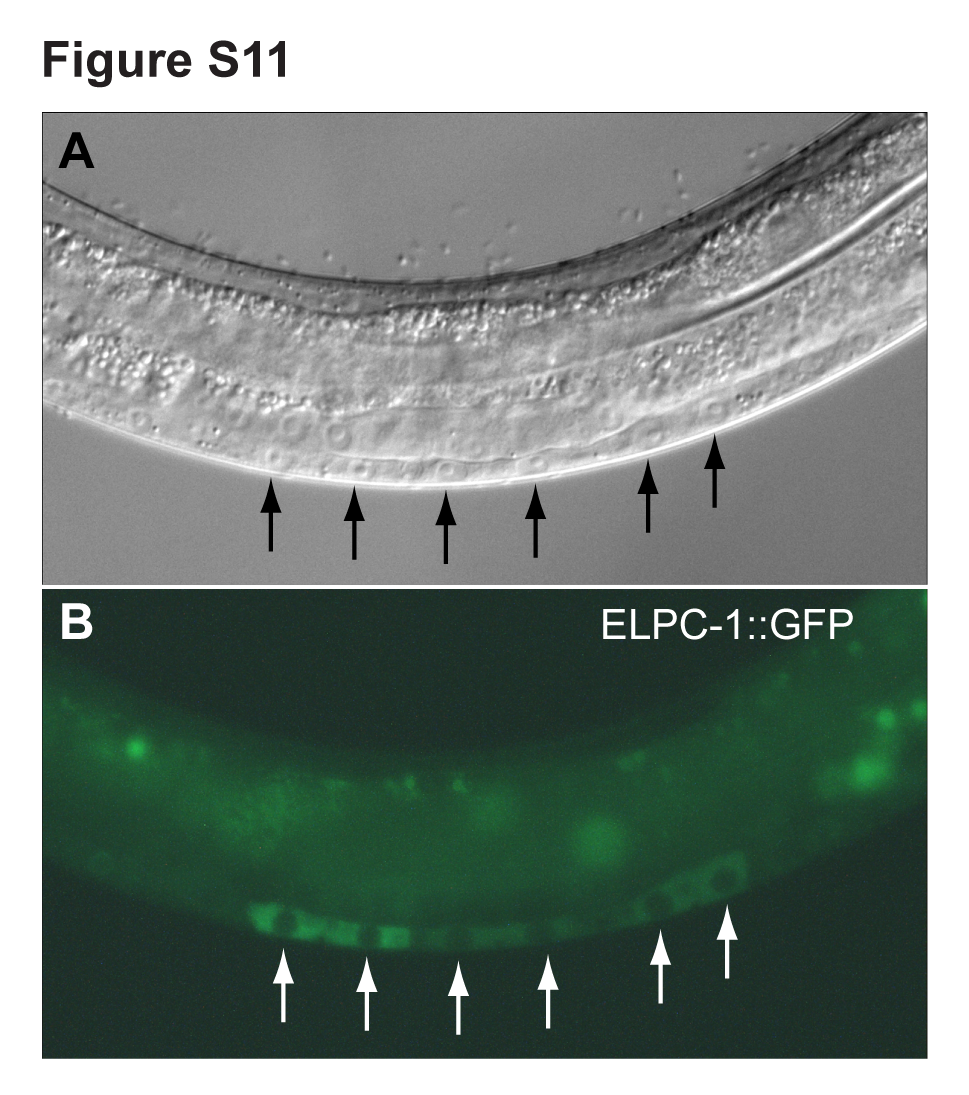

Supplement: Figure S11 — ELPC-1::GFP is expressed during vulval cell fate specification. Micrographs of an L3 hermaphrodite worm of the genotype elpc-1(tm2149); svEx557[Pelpc-1::elpc-1::gfp] viewed with either Nomarski differential contrast (DIC) (A) or fluorescence (B) optics. The arrows denote the six descendants of P5.p, P6.p and P7.p. (1.58 MB TIF) [file pgen.1000561.s011.tif]
